# The evolution of TEP1, an exceptionally polymorphic immunity gene in *Anopheles gambiae*

**DOI:** 10.1186/1471-2148-8-274

**Published:** 2008-10-07

**Authors:** Darren J Obbard, Deborah M Callister, Francis M Jiggins, Dinesh C Soares, Guiyun Yan, Tom J Little

**Affiliations:** 1 Institute of Evolutionary Biology, School of Biological Sciences, University of Edinburgh, West Mains Rd, Edinburgh EH9 3JT, UK; 2 School of Chemistry, Joseph Black Building, King's Buildings, University of Edinburgh, Edinburgh EH9 3JJ, UK; 3 Program in Public Health, College of Health Sciences, University of California at Irvine, 252 Hewitt Hall, Room 3038, Irvine CA 92697-4050b, USA

## Abstract

**Background:**

Host-parasite coevolution can result in balancing selection, which maintains genetic variation in the susceptibility of hosts to parasites. It has been suggested that variation in a thioester-containing protein called *TEP1 *(AGAP010815) may alter the ability of *Anopheles *mosquitoes to transmit *Plasmodium *parasites, and high divergence between alleles of this gene suggests the possible action of long-term balancing selection. We studied whether *TEP1 *is a case of an ancient balanced polymorphism in an animal immune system.

**Results:**

We found evidence that the high divergence between *TEP1 *alleles is the product of genetic exchange between *TEP1 *and other TEP loci, i.e. gene conversion. Additionally, some *TEP1 *alleles showed unexpectedly low variability.

**Conclusion:**

The *TEP1 *gene appears to be a chimera produced from at least two other *TEP *loci, and the divergence between *TEP1 *alleles is probably not caused by long-term balancing selection, but is instead due to two independent gene conversion events from one of these other genes. Nevertheless, *TEP1 *still shows evidence of natural selection, in particular there appears to have been recent changes in the frequency of alleles that has diminished polymorphism within each allelic class. Although the selective force driving this dynamic was not identified, given that susceptibility to *Plasmodium *parasites is known to be associated with allelic variation in *TEP1*, these changes in allele frequencies could alter the vectoring capacity of populations.

## Background

Host-parasite coevolution can take different forms. For example, coevolution can involve repeated selective sweeps, which drives divergence between species while diminishing polymorphism within species [1,2]. Many more immune system genes show evidence of selective sweeps than genes with other functions [3,4]. However, coevolution is also associated with balancing selection, which is of particular interest as it can maintain functionally important polymorphism within species [5-8]. Quantitative genetic studies have revealed substantial genetic variation for infection-related traits in a wide range of organisms (reviewed in [9]). Analyses of DNA sequence polymorphism can provide certain evidence as to whether this is due to balancing selection. For example, the action of balancing selection may be evident in allele frequency distributions or due to the fact that balancing selection promotes sequence differences between alleles [10-13]. However, phenomena such as unexpectedly deep divergence between alleles can have other origins, such as gene conversion.

Analyses of the immunity genes of *Anopheles gambiae*, the primary mosquito vector for *Plasmodium falciparum *in Africa, have, for some time, lagged behind those of the model *Drosophila*, but this is changing. For example, RNAi knockdown studies have now identified many genes which act as antagonists of parasite development, and also genes that act as agonists protecting the parasite from mosquito immune responses [14]. Additionally, major-effect Quantitative Trait Loci that make mosquitoes resistant to *Plasmodium *have been identified in natural *Anopheles *populations [15]. However, although it is clear that phenotypic variation for resistance to malaria is abundant in natural *A*. *gambiae *populations [15,16], it has not yet been precisely determined which *Anopheles *genes explain variation in resistance to *Plasmodium *(or indeed any parasite or pathogen of *Anopheles*). Studies of polymorphism, which can recognise the action of selection and help identify the genes that underlie phenotypic patterns of resistance, are increasing [17-20], although have not yet thrown up any clear candidate targets of parasite-mediated selection.

A key immunity gene identified through functional studies on *An. gambiae *was a thioester-containing protein (*TEP1*)[21,22]. In vertebrates, the TEP family includes the broad spectrum serine protease inhibitors α2-macroglobulins, and complement factors, which are involved in the labeling and destruction of pathogens. Fifteen *TEPs *have been identified in the *An. gambiae *genome, and some, including *TEP1*, are up-regulated upon infection with *Plasmodium bergei *[23], a cause of rodent malaria commonly used as model for the study of human malaria [24]. *TEP1 *is secreted by mosquito hemocytes into the hemolymph, where it is cleaved after septic injury and then binds to pathogen surfaces through the thioester bond. Through this activity, *TEP1 *may be one of the factors that determine vectorial capacity in *An. gambiae*. The knockdown of *TEP1 *in a susceptible strain resulted in a five-fold increase in the number of *P. bergei *oocysts developing in the midgut, while in a resistant strain of mosquito, the knockdown abolished parasite melanisation, thus rendering mosquitoes susceptible [23].

These susceptible and resistant laboratory mosquito strains possess different alleles at *TEP1 *(*TEP1s *and *TEP1r*, originally labeled in the genome annotation as different genes, *TEP1 *and TEP16), but it is unknown if this variation causes the observed differences in resistance. Intriguingly, however, the identity between the *TEP1s *and *TEP1r *deduced amino acid sequences is less than 90% in some regions, including the functional domain that contains many of the key features of the molecule [23,25]. This similarity is exceptionally low for alleles at a single locus, and suggests that the two allelic classes at *TEP1 *are much more ancient than alleles at other loci in *An. gambiae *(or indeed in the majority of animal taxa), as can occur through balancing selection. This study therefore investigated the possibility of balancing selection at *TEP1 *by gathering DNA polymorphism data in African populations of *An. gambiae*. Our data provide a rigorous statistical test for the anecdotal observation that the *TEP1 *locus harbours unusually divergent alleles. We also find evidence of recent selection affecting allelic frequency. However, our data also make it clear that genetic exchange has occurred between the TEP family members, potentially making it difficult to distinguish the effects of selection from those of gene-conversion. Indeed, in the case of *TEP1*, the latter is a more compelling explanation for the deep divergence of alleles at *TEP1*.

## Results

### Genetic exchange between loci

**Figure 1:**
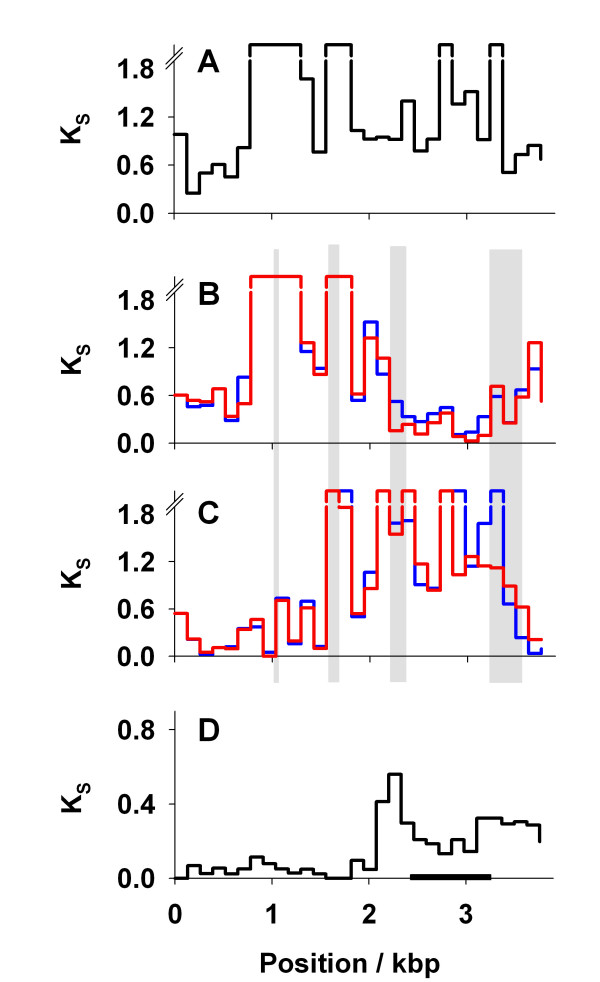
**Evidence for a chimeric origin of *TEP1***. Synonymous site divergence (*K*_*S*_) between *TEP5*, *TEP6*, and *TEP1 s *and *r*, calculated for 30 consecutive windows of coding DNA sequence. (A) Divergence between *TEP5 *and *TEP6*. (B) Divergence between *TEP1 *(*s *in blue, *r *in red) and *TEP6*. (C) Divergence between *TEP1 *and *TEP5*. (D) Divergence between *TEP1s *and *TEP1r*. In (D), note that the region of high divergence between *TEP1s *and *TEP1r *covers sites 2100–3700 and the TED domain is shown as a black bar, and that in (B and C) *TEP1 *is most similar to *TEP6 *at sites 100–1500, but is most similar to *TEP5 *at sites 2000–3200 bp; specifically in the region 100–1500 the divergence between TEP6 and TEP1 is *K*_*S *_= 0.87 (95% bounds 0.70–1.10), but in the region 2000–3200 this divergence drops to *K*_*S *_= 0.37 (0.30–0.46) which differ significantly (*p *< 0.001 [37]). Note also that within the divergent region, TEP1r is consistently more similar to TEP6 than is TEP1s (red line vs. blue line); for example the divergence between TEP1r and TEP6 between sites 2250 and 3250 is *K*_*S *_= 0.15 (0.10–0.20) but between TEP1s and TEP6 is *K*_*S *_= 0.27 (0.20–0.35). Regions in which the MaxChi test suggests there is significant evidence (*p *< 0.05) for recombination breakpoints between *TEP1 *and *TEP5 *or *TEP6 *are shown as grey bars. The graph has been truncated when *Ks*>2

Alignments between the coding sequences of *TEP1 *(AGAP010815) and TEP's 5 and 6 (annotated as *TEP17 *(AGAP010814) and *TEP18 *(AGAP010813) respectively in Ensembl release 49, March 2008) clearly show the level of divergence between loci is not consistent along the length of the gene (Figure 1). Specifically, divergence (*K*_*s*_: the number of synonymous substitutions per synonymous site) between *TEP1 *and *TEP5 *or *TEP6 *varies from *K*_*S *_>1 in some regions (i.e. higher than can be reliably estimated using a simple model of substitution) to *K*_*S *_~0.03 in others (on a par with typical divergence between alleles). This suggests that different parts of the gene may have experienced different evolutionary histories. In particular, *TEP1 *appears to be a chimera of a TEP5-like gene (Figure 1C; *ca *1 – 1.5 Kbp) and a *TEP6*-like gene (Figure 1B; *ca*. 2 – 3.2 Kbp). Consistent with this, the MaxChi test [26] identifies four regions of *TEP1 *that show significant evidence of recombination with *TEP5 *and/or *TEP6 *(recombination breakpoints shown as grey boxes, Figure 1).

Across the region of high divergence between *TEP1s *and *TEP1r *(Figure 1D; from site 2100 to the 3' end of the coding DNA sequence) both alleles show high similarity to *TEP6 *(Figure 1B). However, *TEP1r *is consistently more similar to *TEP6 *than is *TEP1s *(red vs. blue lines, Figure 1B). Indeed, all haplotypes in the *TEP1r *class share a region of *ca*. 320 bp within the TED domain (Figure S1) that shows significant evidence of recombination with *TEP6*. Divergence (all sites) between *TEP1r *and TEP6 in this region is only 3.2% (95% bounds by simulation 1.2–5.5% using K-estimator) but divergence between *TEP1s *and *TEP6 *is three times larger, at 14.6% (10.4–19.5%). This suggests a more recent shared ancestry for the *TEP1r *and *TEP6 *sequences in this region. Thus, although *TEP1 *appears to be a *TEP5/TEP6 *chimera due to gene conversion, it seems that conversion events with *TEP6 *have occurred more recently for the *TEP1r *allele than they have for the *TEP1s *allele.

For a very small minority of individuals for which we sequenced the TED domain appeared to be a recombinant between *TEP1r *and an unidentified TEP6-like gene (Figure S2, lower panel). This suggests gene conversion into *TEP1 *from yet another locus.

### Genetic exchange between *TEP1s *and *TEP1r*

Although the high divergence between *TEP1s *and *TEP1r *suggests that recombination between them is rare, if the sequences are allelic then some evidence of recombination might be expected. Within the region where *TEP1s *and *TEP1r *are highly divergent, we identified six recombinant sequences between the two allelic classes (Figure S2, Genbank Accessions EU881745–EU881867), although all but one of the recombinants were at low frequency.

**Figure 2:**
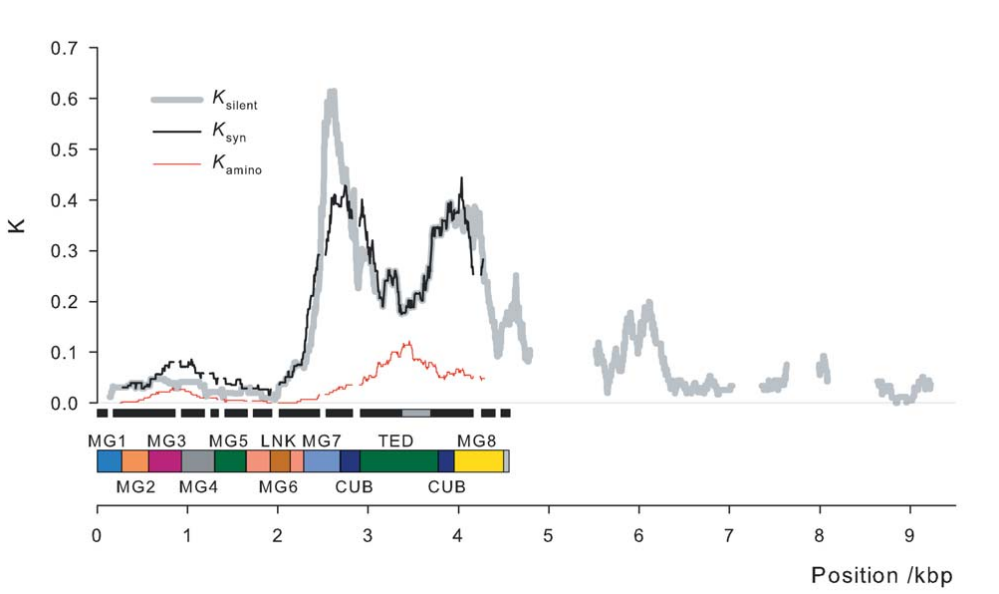
**Divergence between *TEP1s *and *TEP1r *alleles**. The proportion of sites that differ between the *TEP1s *and *TEP1r *alleles are plotted against position for all silent sites, synonymous sites, and non-synonymous sites). Genomic sequence spanned by the macroglobulin domains 1–8 (MG), a linker (LNK), a β-sheet and the thioester-containing domain (TED) are marked below the x-axis. Also marked are exons (solid black bars) and a region of putative gene conversion from TEP6 (grey bar, see main text for details). For zero to 5 kbp, 14 *TEP1s *and 15 *TEP1r *haplotypes were used; for 5 kbp to end, 6 *TEP1s *and 5 *TEP1r *haplotypes were used. Moving windows were 100-site for silent and synonymous sites, 300-site for non-synonymous sites and gaps in *K*_*silent *_correspond to regions with large indels and/or no discernable alignment between TEP1s and TEP1r haplotypes.

The one high-frequency recombinant occurred only in the Cameroon population sample, where it represented 15 of the 24 sequenced haplotypes. In these sequences, an 80 bp region in the *TEP1r *TED domain appears to have been copied into the *TEP1s *allele (location marked by a white bar in Fig S1). It is interesting to note that: (1) this region nests within the putative gene conversion from *TEP6*, such that this sequence appears to have spread from *TEP6 *throughout the *TEP1r *allelic class into *TEP1s*; and (2) this region includes the peak of maximum amino acid divergence between allelic classes (red line, Figure 2)

### Patterns of genetic diversity at the *TEP1 *locus

Our results confirm that overall genetic diversity in *TEP1 *is high (*π*_*S *_> 10% for *An. gambiae *'Mbita', Table 1), and that amino acid diversity is exceptionally high (*π*_*A *_~4%, Table 1)[17]. As described previously [23,25], high diversity in *TEP1 *results from co-occurrence of the divergent *TEP1s *and *TEP1r *allelic classes, and that amino acid differences between the two allelic classes are likely to cause functional differences [27](see also Figure S3, additional file 1). Our data additionally show that the region of high divergence covers the 3' half of the coding sequence and extends almost 2 Kbp into 3' flanking DNA (Figure 2). Silent site divergence (*K*_*S*_) between these allelic classes exceeds 40% in some places, and is approximately 20% in the TED domain, where amino acid divergence (*K*_*A*_) reaches >10% (Figure 2). The co-occurrence of these highly divergent allelic classes gives a significantly positive Tajima's *D *statistic (Table 1) and results in unusual haplotype structure (Table 2): given the number of segregating sites, there are significantly too few haplotypes and a most-common haplotype that is significantly too common. Thus, the distribution of genetic diversity between the *TEP1s *and *TEP1r *allelic classes is incompatible with a standard neutral model of molecular evolution.

**Table 1:** DNA sequence polymorphism summary statistics for *TEP1*

	n^b^	length^c^/bp	*N* ^d^	*S* ^e^	π_A_^f^	π_S_^g^	θ_A_^h^	θ_s_^i^	Taj *D*^j^	Fu&Li *D*^j^	Walls Q
**TED domain**											

*An. arabiensis *(all^a^)	35	661	47	32	3.87	8.92			2.07*	0.89	0.68***
*TEP1s*	11	828	5	7	0.31	0.91	2.67	1.27	-1.16	-0.71	0.00
*TEP1r*	24	839	6	6	0.20	0.44	0.25	0.85	-1.45	-1.71	0.42
Mbita (all^a^)	46	736	44	38	3.81	10.58			2.93**	1.90**	0.76***
*TEP1s*	34	736	2	8	0.15	1.70	0.09	1.19	1.5	1.40**	0.2
*TEP1r*	12	840	2	1	0.15	0.09	0.10	0.17	-1.14	-1.33	0.00
Cameroon (all)	24	840	11	9	0.78	1.85	0.45	1.26	1.47	1.37	0.90***
*TEP1s*	9	840	2	6	0.17	1.58	0.11	1.15	1.57	1.37	1.00***
*TEP1s/r*	15	840	0	0	0.00	0.00	0.00	0.00	..	..	..
Burkina Faso (all r)	12	840	0	2	0.00	0.34	0.00	0.35	-0.05	-0.37	0.00

**Region of *TEP1s*/r divergence**											

*An. arabiensis*											
*TEP1s*	7	2292	8	14	0.22	1.15	0.21	1.19	0.18	0.05	0.16
*TEP1r*	20	2316	8	15	0.06	0.48			-1.61	-2.23	0.25
Mbita											
*TEP1s*	19	2299	10	25	0.17	1.81	0.18	1.48	0.80	1.19	0.14
*TEP1r*	10	2320	4	7	0.07	0.32	0.09	0.51	-1.57	-1.63	0.18

a – samples were not at random with respect to allelic class, and thus do not exactly reflect natural allele frequencies

b – number of haplotypes sequenced

c – analyzed length of sequence

d – non-synonymous segregating sites

e – synonymous segregating sites

f – average pairwise genetic diversity at non-synonymous sites

g – average pairwise genetic diversity at synonymous sites

h – Watterson's estimate of 4 Nμ per site for non-synonymous sites

i – Watterson's estimate of 4 Nμ per site for synonymous sites

j – Tajimas D statistic and Fu & Li's D statistic calculated for synonymous sites only

k – Walls Q statistic

**Table 2:** Haplotype frequency tests

	*n*	*S*	θ	*K*	*M*	Haplotype configuration
Arabiensis^a^	19	79	22.6	7*	7*	(4,1,0,0,0,1,1,0,0,0,0,0,0,0,0,0,0,..)***
*TEP1s*	9	10	3.7	5	3	(3,0,2,0,0,0,0,0,0,0,0,0,0,0,0,0,0,..)
*TEP1r*	12	8	2.6	4	6	(2,0,0,1,0,1,0,0,0,0,0,0,0,0,0,0,0,..)
						
Mbita	17	85	25.1	3***	9***	(0,0,0,2,0,0,0,0,1,0,0,0,0,0,0,0,0,..)***
*TEP1s*	24	10	2.7	4	16	(0,0,1,1,0,0,0,0,0,0,0,0,0,0,0,1,0,..)
*TEP1r*	10	2	0.7	2	6	(0,0,0,1,0,1,0,0,0,0,0,0,0,0,0,0,0,..)
						
Cameroon	24	20	5.4	4*	15*	(0,1,1,1,0,0,0,0,0,0,0,0,0,0,1,0,0,..)***
*TEP1s*	9	8	2.9	3	4	(0,1,1,1,0,0,0,0,0,0,0,0,0,0,0,0,0,..)
*TEP1s/r*	15	0	0.0	1^b^	15^b^	(0,0,0,0,0,0,0,0,0,0,0,0,0,0,1,0,0,..)^b^

*n *is the number of haplotypes sequenced, *S *the number of segregating sites, θ Watterson's estimate of 4 *Nμ *per gene, K the number of haplotypes, M the frequency of the commonest haplotype. The 'Haplotype Configuration' is a vector (n_1_, n_2_, n_3_, ..., n_i_) giving the number of distinct haplotypes that appeared *i *times in the sample (see Innan *et al *2005). For example, the first haplotype configuration (4,1,0,0,0,1,1,...) has four haplotypes that appeared once each, one that appear twice, none that appeared three, four or five times, one that appeared six times and one that appeared seven times. ^a^Sampling was not at random with respect to s/r state, ^b^significance not tested. Significance assessed through coalescent simulation is indicated as *p < 0.05, ***p < 0.001.

Within the divergent allelic classes (treated separately), genetic diversity is low (*π*_*S *_~1%, Table 1) – closer to that displayed by other *An. gambiae *loci [17] – and neither Tajima's *D *statistic (Table 1) nor the haplotype configuration statistics (Table 2) identify any significant deviation from neutrality. However, it is notable that genetic diversity is consistently lower and Tajima's *D *(non-significantly) more negative for *TEP1r *than *TEP1s*, suggestive of a smaller long-term effective population size and a recent increase in frequency for *TEP1r*. Most strikingly, in the Cameroon sample, the *TEP1s *allelic class displays normal genetic diversity (*π*_*S *_= 1.6%) but there is no variation at all amongst the *TEP1s/r *putatively recombinant sequences.

### Evidence for selection

Using a McDonald-Kreitman test, we found that there was a significant excess of amino-acid difference between the *TEP1s *and *TEP1r *relative to the levels of polymorphism (Table 3). This suggests that natural selection has driven the adaptive divergence of the protein sequences of the two alleles. Next we tested whether natural selection has changed the frequency of the two alleles relative to each other. Under the null hypothesis that their frequency has been constant through time, the genetic diversity within each allelic class will be proportional to its frequency in the population. However, we found significantly reduced genetic diversity among both the *TEP1r *alleles of *An. arabiensis*, and in the *TEP1s/r *recombinant alleles from the Cameroon population of *An. gambiae *(Table 4). This suggests there has been a recent increase in the frequency of these alleles in these populations, although we cannot with certainty attribute this to selection, rather than a demographic effect.

**Table 3:** McDonald and Kreitman tests of whether natural selection has driven the divergence of the *TEP1s *and *TEP1r *protein sequences.

Region analysed	*n*^a^	Codons^b^	*Ds*	*Ps*	*Dn*	*Pn*	NI^c^	*p*^d^
Whole CDS	29	1287	98	87	76	36	0.53	0.015
5' end (low divergence)	29	417	1	29	2	20	0.345	>0.5
3' end (high Divergence)	29	870	97	58	74	16	0.362	0.001
TED	85	272	30	23	42	12	0.373	0.024
remainder	29	598	65	43	30	10	0.500	0.123

Sequences of *TEP1s *and *TEP1r *were pooled across all populations and both species (*An. gambiae *and *An. Arabiensis*). Tests were conducted separately for regions that show high or low divergence between alleles, as well as separately for the TED region. The test is accomplished by comparing divergence and polymorphism at synonymous sites (*Ps *and *Ds*, respectively) to divergence and polymorphism at nonsynonymous sites (*Dn *and *Pn*, respectively)

a – Total number of haplotypes

b – Analysed codons

c – Neutrality Index

d – p-value 2-tailed fishers exact test

**Table 4:** Test for a recent change in allelic frequency based on the distribution of segregating sites between allelic classes.

Sample	Allelic class	Class Count^a^	Sequenced Haplotypes^b^	Observed Segregating sites^c^
*An. arabiensis *(TED)	S & R	11 s | 33 r	11 s | 24 r	12 | 12^#^
*An. gambiae *'Mbita' (TED)	S & R	32 s | 13 r	27 s | 12 r	10 | 3^*ns*^
*An. gambiae *'Cameroon' (TED)	S & R conversion S & R	9 s | 15 sr	9 s | 15 sr	8 | 0 *
*An. arabiensis *(all)	S & R	11 s | 33 r	7 s | 20 r	22 | 23*
*An. gambiae *'Mbita' (all)	S & R	32 s | 13 r	19 s | 10 r	35 | 11^ns^

^a ^The observed frequency of the *TEP1s *and *TEP1r *classes; ^b ^the number of haplotypes sequenced from each allelic class, ^c ^the total number of synonymous and non-synonymous segregating sites observed in that allelic class. Significance was assessed using simulations under a neutral model in which the frequency of each allelic class is constant, and proportional to the observed frequency (See main text, and Stahl et al 1999)

^*ns *^p > 0.05; ^# ^p < 0.1; * p < 0.05,

## Discussion

The *An. gambiae *immunity gene *TEP1 *is exceptionally polymorphic in a region than includes the key functional features of the protein, and patterns of diversity are incompatible with a neutral model of evolution (see also [28]). Localised amino acid divergence (in particular at exons 7–10, Figure 2) between the *TEP1 *alleles ranged up *ca*. 10%, which is reminiscent of cases of balancing selection such as human MHC class I alleles which show up to 19% amino acid divergence between alleles [10]. Assuming the *Drosophila *[29] synonymous substitution rate of 1× 10^-8 ^site^-1 ^year^-1^, the divergent part of the *TEP1s *and *TEP1r *coding alleles shared a common ancestry of about 15 million years ago, which is truly exceptional for alleles at a single locus.

A key question then, is whether the alleles evolved their divergence *in situ *(presumably via balancing selection), or did the sequences diverge at separate loci, and then become allelic due to gene conversion? Our data support gene conversion between *TEP1 *and other loci as a likely origin for high divergence. *TEP1 *as a whole may be a chimera (Figure 1B and 1C) produced from *TEP5*-like and *TEP6*-like genes. The divergence between the 's' and 'r' alleles may then represent *TEP1*-*TEP6 *gene conversion at different time points. Specifically, although the entire *TEP1 *divergent region is remarkably like *TEP6*, *TEP1r *is more similar to *TEP6 *than is *TEP1s*, and thus *TEP1r *appears to have been a more recent conversion. We can roughly estimate the time since this conversion based on the observed number of segregating sites (mutations occurring since the conversion [30]) over the ~2.3 kbp region of allelic divergence, assuming a star shaped genealogy of each allelic class [30]. In 10 *An. gambiae *individuals we observe that seven of 485 synonymous sites are variable, which (taking the *Drosophila *mutation rate given above) suggests they shared a common ancestor approximately 150 thousand years ago.

Whatever the origins of high divergence between the *TEP1s *and *TEP1r *allelic classes, the locus still appears to be the target of natural selection. Specifically, the *TEP1s/r *recombinant allele has significantly reduced diversity in Cameroon, indicating that natural selection has caused it to recently increase in frequency. Indeed, this recombinant allele shows no diversity whatsoever, while this is not so for its counterpart within the same population. A similar pattern was found in *An. arabiensis*, where there is a significant reduction in the genetic diversity of the *TEP1r *allele. McDonald-Kreitman tests also revealed an excess of non-synonymous divergence between alleles [31], which might indicate that positive selection has driven the divergence of alleles.

Other TEP genes from *Drosophila *and the crustacean *Daphnia *[32,33] have recently been shown to evolve rapidly under positive selection. This growing body of evidence suggests that TEP genes may be key sites of host-parasite co-evolution, and are subject positive selection (which is focused in the bait-like region corresponding to the bait region of α-macrogloblins and the anaphylatoxin fragment of the complement protein C3 in vertebrates). Our *Anopheles *study focused on the TED-like region, but the corresponding TED-like domain in *Drosophila *appears to be fairly conserved, suggesting that its functional significance varies between insects, and possibly that its function may be dependent on the types of host and parasite molecules interacting. Further work on polymorphism and divergence at TEP genes seems an exceptionally promising path to gain insight into the tempo and mode of evolution at immune system genes as well as parasites strategies to overcome host defenses.

In conclusion, although we find evidence that the origin of divergent *TEP1 *allelic classes is due to gene conversion and not balancing selection, they may still represent functionally relevant polymorphism. That natural selection has increased the frequency of one allele relative to the other suggests that there are important functional differences between alleles, although the selective force driving this change has not been identified. What ever the cause of allele frequency changes, such evolution could alter vectoring capacity, because different *TEP1 *alleles are established to alter susceptibility [21,23,27], at least to some *Plasmodium *species. Further functional studies of differences between TEP alleles remain desirable, in particular if the alleles could be studied in a randomized set of genetic backgrounds and if studies could include both infection-related traits and other general measures of fitness.

## Methods

### Sample origin

*An. gambiae *individuals were collected from three sites: Mount Cameroon region (Cameroon, provided by S. Wanji, University of Buea), Burkina Faso (Koubri village (12°11'54 N; 1°23'43 W), Mbita (Suba District, Western Kenya, provided by H. M. Ferguson, University of Glasgow, UK). *An. arabiensis *individuals were collected from two sites: Tanzania (Ifakara, provided by H. M. Ferguson, University of Glasgow, UK) and Mbita (Suba District, Western Kenya). Species status of all specimens was verified by diagnostic PCR [34]. We did not distinguish between M and S molecular forms of *An. gambiae *s.s Although differentiation between M and S molecular forms might in principle compromise parts of our analysis, there is strong evidence that differentiation is very low in this region [35].

### PCR and sequencing

Genomic DNA was extracted from single mosquitoes using the QIAgen DNeasy kit (QIAgen Ltd., UK). For short fragments, PCR was performed using BioTaq (Bioline, London, UK) and for longer fragments using the Expand Long Template kit (Buffer 2; Roche Applied Science, Mannheim, Germany). Two different sequencing strategies were adopted, according to fragment length and allelic state. Firstly, for short regions comprising the Thioester Domain (TED) only, PCR products were amplified without reference to allelic class (i.e. *TEP1s *vs. *TEP1r*), and cloned using TOPO Kits (Invitrogen Ltd, Paisley, UK). At least six clones were sequenced from each PCR product to ensure both alleles were identified. Secondly, allele-specific primers for *TEP1s *and *TEP1r *were developed to allow: (1) screening for allelic class; and (2) sequencing of single haplotypes from s/r heterozygous individuals. These allele-specific primers were either paired with TED primers (giving overlapping allele-specific fragments for this domain) or, for longer fragments, with primers placed 5' in exon 2, and *ca*. 2.7 kb 3' from the end of the *TEP1 *CDS. For several individuals we were unable to amplify the ~5.5 Kb fragment, and instead used additional allele-specific primers placed ~500 bp 3' of the CDS to amplify this region in two parts. Individuals could therefore be identified as s/r heterozygotes using short amplified regions, and subsequently targeted for long allele-specific PCR and sequencing. In total we amplified 102 haplotypes covering the TED domain (0.84 Kbp), 29 covering the majority of the CDS (4.8 Kbp), and 11 haplotypes extending ~4 kb into non-coding DNA (8.7 kb)(Figure 2).

Before direct sequencing of PCR products, unincorporated dNTPs and primers were removed by incubation with Exonuclease I (New England Biolabs) and Shrimp Alkaline Phosphatase (Amersham). Cloned fragments were sequenced directly from plasmids after purification with the QIAgen Plasmid Mini Kit (QIAgen Ltd., UK). Sequencing was performed in both directions using BigDye™ reagents (v3.1, Applied BioSystems) and an ABI capillary sequencer. PCR and sequencing primers for each amplified region were designed from the published genome sequence of *An. gambiae *[36] and sequences are given in additional file 1 (Table S1). All sequence chromatograms were inspected by eye to confirm the validity of variable sites, and assembled using SeqManII (DNAstar Inc., Madison USA). All sequences have been submitted to GenBank as an aligned set: sequence accession numbers span the range EU881745–EU881867.

### Recombination and gene conversion

*TEP1 *is part of a recently expanded gene family in *Anopheles *mosquitoes [25], and occurs in close physical proximity to other TEP genes (e.g. TEP5 and TEP6, cytological band 39C on chromosome 3L). Additionally, the *TEP1s *and *TEP1r *allelic classes are known to be more divergent in some regions of the gene than others [23,25]. These observations led us to hypothesize that gene conversion and/or recombination, either between *TEP1s *and *TEP1r *allelic classes, or between neighboring genes, may have played a role in *TEP1 *evolution. We therefore examined our *TEP1 *s/r sequences, along with genomic sequences at the neighbouring genes TEP5 and TEP6, for evidence of genetic exchange.

To visually identify potential regions of exchange, we used K-estimator [37] to estimate genetic divergence (under the Kimura 2-parameter model) at synonymous sites between *TEP5*, *TEP6*, *TEP1s*, and *TEP1r*, in 30 consecutive blocks across the coding sequence. To examine physical distribution of genetic divergence between *TEP1s *and *TEP1r *allelic classes in more detail, and for plots of diversity and divergence using multiple alleles, we used DNAsp [38,39] to estimate average genetic divergence in a sliding-window analysis at synonymous, non-synomymous, and non-coding sites. To statistically identify regions of genetic exchange, we used the MaxChi test [26] as implemented in the R statistical computing language [40]. This test uses a sliding window analysis, focusing on a series of points along the alignment. For each focal point (window center) a chi-square statistic is calculated to compare the proportion of matching sites to the left with the proportion of matching sites to the right, such that a recombination event at the focal point would lead to a high statistic. The maximum chi-square statistic observed is a summary of the evidence for recombination at the focal point, and significance of the observed chi-square statistic is assessed by a permutation test.

The processes that promote genetic exchange, such as high similarity and close physical proximity, may compromise automated genome assembly and annotation. Some of the current *TEP1 *gene models in the *Anopheles gambiae *genome may be insufficiently robust to reliably detect gene conversion and recombination. In particular, the current Ensembl assembly (Ensembl 49, March 2008) only identifies a small part of TEP6 (there labeled TEP18), and gives a large stretch of TEP5 (there labeled TEP17) as being 100% identical to *TEP1 *– which is highly unlikely, given normal background levels of genetic diversity. We therefore sought to obtain improved models of *TEP5 *and *TEP6 *by manually creating new assemblies from trace files generated by the on-going sequencing of the S-molecular form of *An. gambiae *s.s. (Ewen Kirkness, JCVI, and [41]) Our manually-curated assemblies for these two genes have are supplied in additional file 1 (Figure S4), and identifiers for the trace files used are given in additional file 1 (Table S2). Hereafter, we refer to *TEP5 *and *TEP6 *as we have derived them from the S-form traces. Note that although our inference of 1:1 correspondence between our 'TEP5' and 'TEP6' sequences and those currently annotated in the genome may prove incorrect (e.g. as more informative genomic and expression data become available), this does not affect any inferences regarding genetic exchange.

### Genetic diversity

Using DNAsp we calculated total genetic diversity (*π*), Watterson's estimate of *θ*, Tajima's *D *[42], and Fu & Li's *F *[43] for synonymous sites. This was done for combined *An. arabiensis *data, and separately for *An. gambiae *samples from Mbita, Cameroon, and Burkina Faso. Statistics were calculated across all sequences, and for *TEP1s *and *TEP1r *allelic classes separately; and where sampling permitted, for two different amplified regions: (1) a short fragment covering only the TED domain (~800 bp); and (2) a longer fragment (with smaller sample size) covering the entire region of high divergence between *TEP1s *and *TEP1r *allelic classes (~2.2 Kbp). Haplotype statistics (number of haplotypes, frequency of the commonest haplotype, haplotype configuration) were calculated using DNAsp, and their probability under a neutral model of evolution assessed by coalescent simulation as implemented in Haploconfig [44], conservatively assuming no recombination within loci. Haplotype statistics were calculated across all sampled *TEP1 *alleles, and separately for the *TEP1s *and *TEP1r *allelic classes.

### Tests of neutrality within allelic classes

Firstly, to test for a non-neutral rate of protein evolution we used a test directly analogous to that of McDonald and Kreitman (MK) tests [45], but applied to the divergence between the *TEP1s *and *TEP1r *allelic classes rather than to divergence between species. MK tests infer selection from an excess of amino acid substitution between lineages, assuming that synonymous sites are selectively neutral (or close to neutrality) and that polymorphic non-synonymous sites are close to neutrality. If the analysis is restricted to sequences that display no evidence of recombination, and if selective constraint is equal for the two allelic classes, the assumptions of the MK test are met by highly divergent groups of alleles such as *TEP1s *and *TEP1r*, and the MK test can be used to ascribe divergence between allelic classes to adaptive evolution. MK tests were performed with DNAsp.

Secondly, to test for a non-neutral level of genetic diversity within the TEP1s and TEP1r allelic classes, we applied a test very similar to that of Stahl et al [13]. Our aim was to test whether one allelic class displays significantly reduced genetic diversity compared to the other, given their relative frequencies and our sample sizes. If it does, then this suggests that this class may have recently increased in frequency relative to the other, because increases in frequency are expected to be accompanied by a loss of diversity. Following Stahl *et al *[13] we simulated independent neutral coalescent trees for each allelic class using 'ms' [46], according to the number of haplotypes sequenced for that class, but recording only the total tree length (in units of 4*N*_*e *_generations). These trees were then scaled by the estimated relative effective population size of each allelic class, based on its sampled frequency. Assuming constant effective population sizes for each allelic class, it is expected that the fraction of segregating sites seen in each sample of alleles will be proportional to the (scaled) tree size for that sample. Deviations from this expectation suggest that the allelic classes have changed in frequency relative to each other. Our approach differs from Stahl *et al *[13] in two minor ways: (1) the number of analysed haplotypes in each class can be independent of that class's frequency, since for our data the former comes partly from allele-targeted sequencing and the latter from PCR-assay; and (2), we account for the variance in estimates of class frequency associated with finite sample size by assuming the class frequency (i.e. the proportion that are TEP1r) follows a beta distribution defined by the observed numbers in each allelic class. As with similar analyses [13], in addition to the effect of selection, deviations from this model could be caused by demographic factors such as population size fluctuations and population admixture.

## Authors' contributions

DJO performed all long/allele-specific PCR and sequencing, and all statistical analyses. DMC performed all other sequencing and cloning. FMJ helped design the sequencing scheme and provided advice and support for the analysis. DCS performed the structural modeling. GY helped design the overall project, and provided specimens and support during fieldwork. TJL conceived the project, and TJL and DJO wrote the manuscript with contributions from all the other authors.

## Supplementary Material

Additional file 1 Supplemental data. Click here for file
