# Supplementary material for: The evolution of TEP1, an exceptionally polymorphic immunity gene in Anopheles gambiae
Source: BMC Evol Biol. 2008 Oct 7;8:274. doi: 10.1186/1471-2148-8-274 (PMC2576239; doi:10.1186/1471-2148-8-274)
Supplement: Additional file 1 — Supplemental data. [file 1471-2148-8-274-S1.doc]

**Supplementary data:**

***Table S1.*** PCR and sequencing primers for each amplified region of TEP1 (designed from the published genome sequence of *An.* *gambiae* [1])

31F GCCAACCAGGAATACACTCTGGT

425sF GATCGTGCTGGACACGGAG

531sR CTCGAACACACCGGCATACAG

1220sF CTCGAGCTGCAACCAAGTGAG

2089sF GCTTGGGTCTGTTCGCCAGG

2474sF GTATATAGCCGATGTGACGCTG

2996F CACGGTCATCAAGAACCTGGAC

3057sR GTTGGGAACAAATTTGACCAT

***r_3360F*** GAC**A**AG**G**CCGGT**GC**AG**A**GT**AT**

***s_3359F*** TGAC**G**AG**A**CCGGT**AA**AG**T**GT**GG**

***r_3725R*** TCAT**G**ACCGGTATACCGTC**TGTG**

***s_3788R*** ACAAACGTGTCTTGCGTGa**GT**

3853R TGAACGGTGTAGTCGTTTCGGG

3851sF GTCCCGAAACGACTACACCG

4203sR GCTCGCTGATCGGATTGCG

4336sF TTACGGTGGCACTTCAGTCG

***r_4985F*** **CGCTGTTCCGATTCCAGATCTTCG**

***s_4994F*** **CTTGGTTGCGATCAGAAGCTGCA**

5310sR TAGACATTTCCAGCGCAACTCC

6325sF CATCCGCAGCACCTTCTTACAG

6456R CGTGCCGGACCTCTATACG

6913sF CATTTGCTGTGGAACAGTCACTGC

7445sF CGTCGAGGCAATTGGTCCATTG

7445sR CAATGGACCAATTGCCTCGACG

7888sR CACAAGGCATCGAATCGCTATTCCAC

8785R TGCGTGCATGGACAGGTTACG

**Notes on primer names**

- Numbers refer to the approximate location of the 5'- base of the binding site, labeled from the start of Exon 2 in the TEP1r allelic class. Positions are approximate, depending on alignment and indels.
- ‘F’ indicates forward, i.e. from 5' to 3' with respect to the coding sequence (sequences are given 5'-3', as for synthesis)
- ‘R’ indicates reverse, i.e. from 3' to 5' with respect to the coding sequence (sequences are given 5'-3', as for synthesis)
- ‘s’ indicates the primer was primarily used only as an internal sequencing primer for longer PCR fragments
- Those marked in ***bold italic*** are specific to the allelic class indicated, and successfully distinguish between them. In the primer sequence, **bold** indicates a mismatch to the other allelic class, lower case indicates a mismatch to both classes. Note that ***r_4985F*** and ***s_4994F*** are in a region with no discernable alignment between the two allelic classes

Table S2

**Trace files used to build hypothetical TEP5 coding region**

1101751070418.scf

1101745000869.scf

1102478017916.scf

1101705479290.scf

1101854559564.scf

1101721870189.scf

1102478684166.scf

1102478765117.scf

1101733438756.scf

1101733739656.scf

1101705523849.scf

1102478072755.scf

1101693360750.scf

1101693832540.scf

1101854415104.scf

1101751456716.scf

1101751246977.scf

1101721758656.scf

1101705176826.scf

1101718138055.scf

1101685072892.scf

1101671988995.scf

1101555145390.scf

1101751241596.scf

1101733862120.scf

1101689015902.scf

1102478840185.scf

1101705540734.scf

1101733985446.scf

1101693902845.scf

1101537682304.scf

1101854103883.scf

1101854088702.scf

1101733268067.scf

1101693373801.scf

1101693422851.scf

1101733101853.scf

1101758015792.scf

1101733893477.scf

1101955178083.scf

1101685025254.scf

1101751464704.scf

1101751792300.scf

1101693812013.scf

1101751071796.scf

1101693544363.scf

1101751399088.scf

1102478701238.scf

1101671269884.scf

1102478591597.scf

1101854144203.scf

**Trace files used to build hypothetical TEP6 coding region**

1101733140594.scf

1101693658995.scf

1101705404247.scf

1101721702793.scf

1101751085236.scf

1101705480066.scf

1102478832723.scf

1101758029167.scf

1101751746000.scf

1101671576754.scf

1101854459301.scf

1101705198207.scf

1101733878871.scf

1101671318896.scf

1101718123048.scf

1101733511583.scf

1101537976034.scf

1101758001480.scf

1101692157214.scf

1102140199698.scf

1101733443954.scf

1101692170673.scf

1102478713536.scf

1101751687485.scf

1101740075366.scf

1101721510771.scf

1101854156672.scf

1101733142886.scf

1101854142421.scf

1101751181575.scf

1101671037382.scf

1101854534336.scf

1101733304607.scf

1101537748194.scf

1101733882911.scf

1101671370485.scf

1101671955422.scf

1101751518098.scf

1102141647902.scf

1101685083486.scf

1101751749514.sc


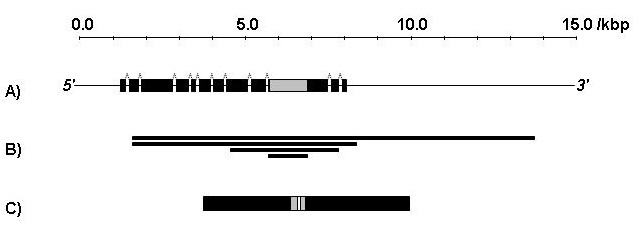


Figure S1: The *TEP1* region

(A) Intron structure of *TEP1* coding sequence with exons marked in black, and the TED domain in grey. (B) The four different regions sequenced (see methods). (C) The region of high divergence over which the *TEP1s* and *TEP1r* alleles can be defined (black). A *TEP1* s/r gene conversion events in our dataset (white), sits within an older gene conversion between TEP6 and the *TEP1r* allele (grey).

**Variable sites**


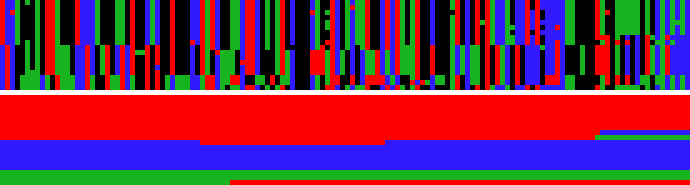


**s**

**r**

**Haplotypes**

*****

*****

*****

Figure S2: Haplotype structure in the TEP1 C3d domain

Upper panel: variable sites in the C3d domain for common and putatively recombinant TEP1 haplotypes (A:green, G:black, T:red, C:blue). The two gene conversions between TEP1s and TEP1r alleles are easily identifiable, as are putative gene conversions with a TEP6-like locus. Lower panel: the same haplotypes colour-coded to emphasise recombinant haplotypes between TEP1s (red), TEP1r (blue) and a TEP6-like sequence (green). The three sites of putative recombination between TEP1s and TEP1r (marked with asterisks) are *p*<0.05 using the MaxChi test with a window size of 20.

**Figure S3: Location of variant amino acids mapped on the crystal structure of TEP-1r.** Pymol (http://www.pymol.org; DeLano Scientific LLC, CA, USA) cartoon schematic representation of the TEP-1r crystal structure [2]. Abbreviations used: macroglobulin = MG; thioester-containing domain = TED; first found in Complement C1r/C1s, Uegf, Bmp1 domain = CUB; linker = LNK; anchor region = ANK. Individual domains colored differently and labeled: MG1 (teal), MG2 (light orange), MG3 (hot pink), MG4 (steel grey), MG5 (dark green), MG6 (peach), MG7 (cyan), MG8 (yellow), TED (green), CUB (dark blue), LNK (red), ANK (grey). Secondary structure elements were assigned by default settings in PyMol. Pairwise comparison between examples of the TEP variants reported in the current study are shown as follows: **(A)** the ‘resistant’ and ‘susceptible’ alleles from one *Anopheles gambiae* individual from Kenya (EU881826 vs EU881827); **(B)** the ‘susceptible’ and an ‘SR’ recombinant allele from one *Anopheles gambiae* individual from Cameroon (EU881778 vs. EU881777); **(C)** the ‘resistant’ and ‘susceptible’ alleles from one *Anopheles arabiensis* individual from Kenya (EU881836 vs. EU881837); and **(D)** the ‘resistant’ and a different potentially recombinant allele from one *Anopheles arabiensis* individual from Tanzania (EU881852 vs. EU881853). The location of variant amino acid residues are highlighted as black spheres. For clarity, only the positions of their equivalent Cα-atom are shown on the structure of TEP-1r. The critical residues involved in formation and catalytic activation of the thioester bond (Cys838, Gln841 and His951) are shown in red spheres within TED. Note no gaps exist between the sequences above and the sequence for the TEP-1r crystal structure. Majority of the variant amino acid residues occur within TED - some of these are present in the vicinity of the thioester and may alter its functionality. Some of the other variants within TED are located in proximity to, or directly at the interface between TED and MG2, MG8 or the CUB domains; these variants may cause a local structural perturbation or alter stability of the intra-molecular domain-domain arrangement within the intact protein.

**Figure S4.** Manually-curated assemblies for TEP6 (named *TEP18* in Ensembl) and TEP5 (named *TEP17* in Ensembl). For comparison, the *TEP1* CDS and current genome assembly are included. Sequences are presented aligned in FASTA format.

TEP6 (Ensembl TEP 18)_region from traces

TTAACAACATGCCCGTCATGGGTTCAAGGCCCGAATAGACCGTGCCCCCATACGTAGGAC

TGACTATCCTGCTATGGTAACAATAAGTCACTGAAAGCCAAGCTCACTTCACTAGTTTGT

ACTGGCAGGCCTTGACCGACAGCGGTTGTTGTGCCATAGAAGAAGAAGAAGCTCTCGTCG

GCCTCTGCCACAGGTAATATACAAATTTTTGTTATCGGACGAGTTACGATACCTTTTGAT

GTTTTCAGTGTTACTACCCGTGTCAGCAGTTCACCTCAAAGAACCTGTCGCGACGGACGA

AGCTGGGACTATATAGTACCTATATAGTACCAGTACTCACATACGCCTCTGAGACATGGA

CACTGTCCAAATCTGACGAAGCCCTCTTAGCCGCGTTCGAGAGGAAGATGCTCAGAAGGA

TACTTGGCCCCGTATGTGTGGAAGGACAATGGAGGAGCCGCTATAATGACGAGCTATACG

AGATGTACGGCGACCTCACTGTCGTAC-AGCGTATTAAGCTCGCCAGGCTCCGGTGGGCT

GGCCATGTTGTACGCATGGAAACGGACGACCCAGCCCGTAAAGTCTTTTTAGGCCGTCCA

CAAGGACAGAGGAGGCGTGGTAGGCCCAAATTGAGGTGGCAAGATGGCGTGGAGGCGTCC

GCCATTAAGCCGGGATAACGGACTGGCAGACGAAGGCGCGAGACCGTGAGCGGTTTCGGA

CACTCCTGAGGCAGGCCAAGACCGCAAAGCGGTTGTAGCGCCGGATAAGTAAGTAAGTAA

GTAAGTAAAATCGTTGAAAAACATAACTATCATATTTTAGAAAGGTTCTACAGATAATTT

TTTTAATGATTTTAATAAGGGTTTTTATAACTATTTCTAGGATTCTGAGAGGTTTATAAG

AACAATTATCAACATTTATTTTGTTTACGTATCAAATGTTTTCAAAACAATCTTTGCATC

--TTATGATTATTGTAAAAATACGTATCAAAATATGTAAAAATATTTTTTAGCGTAGAGA

GTATTAGAGTATTTTGTGAATAATGGTTCAAACCTGATTTGATCAAACAAAAGCGACACA

AGGTTGAGGTTTTCAAAGATTTTTTAGCAAAGTCAAAATATTTGCTGCTATTATATACAT

AATTCTCTCACCACTCACTTCACCATCGTTGTTCTCTTAGACCGCTACAAAAGGGCCCAT

AACCGTCTTCATGTAAAGTTTGGTCGTGAACTCGTAAGGTAGTTTATGAGCCCAAATAAG

GGCTGGTTGAATTAGTAATGTGATATTTACATGTACTAGATAACTCCTTGTAATGCCGAT

AAGAATATGTGGAAAGGGTGTTGCAGTAGGACCATTGTAATTGAATATGGTATTCGTCTG

TGTTTTCTCTAAAACATGTATGGGCGATTTGTAGTGGTCTTGGAGGACCGATCTGGTGGT

ACAGTCGTCAACTCGTACGACTTAACAACATGCTCGTCATGGGTTCAAGGCCCAAATAGC

CCGTGCCCCCATACGTTGGACTGACTTTCCTGCTATGGTAACAATAAGTCAAAGAATGCC

AAGCTCACTTCACCAGTGGGTACAGGCAGGCCTTGACCGACAACGGTTATTGTGCCAAAG

AGGAAGAATAAGAGAACATCGATGGTGAAGTGAGAGGTGAGAGGGAAAAAAWTTCAGGCT

AGGATTGCATTTCTTAACTATGTTTGGAACAGCAGCAAATATTTAGATTCTGTTAAAAAA

TCGTTGTTGAGGTTTTTGTGAGCAAGGCATATTTTGCGACCAGAGTTTAGGACTACTCTA

TATTGCTCTAGCAACC-CAGTACTACAAATGATGAACTCTTATAACGCTCTAGGGTACGA

AGTAGATTTTCATATGACAATAAATCAACTAATGACTTGTTTAAGACAGCTTTTCTGAYG

ATTATATAATGTTCGGTGTAGCTAGTTTTATTTTTATAACGCGTCGAATCAATGGTGTCT

GTTTAAAGYTTTAATTAGGTTAAGATTTTTTAGTTCGAATAATGATATTGCTCGACCACT

AAATAAATAATAAACTAATAAATAATAATACTAAATAAAT-TGACAAGGT---TCTCATT

GCCATTTTAAAAATGAATTAAGAAATATTTTCTCCTATTTTGATTGTTTTTGTATTAAAA

AAATCATAACATGCAAATA--ATTTGTGTTTGAAATCTTTTGATACGTAAAACAAATATA

ATTCTG-ATAATTATTCTTATAAAACTCTCTAAACCTTAAAAAACCATTATCGA-AACAC

TTTC--TAAAATCGTTTAAACATTTTTTCTGTAGACTTTT---CTAACTATGTTATTTAT

ATCTTTGAACAATTTTTAATTGCGTCAGTAACCAAACAAAAAAAAAAAGTTTTTCGAAGT

GTTTTTATAGTTATGGTTCGACCATTTTTGTAATAAGTCGTGTAAAAAATTTTAGAACAT

TTTTCATAAAATAAAAGAAGATGTGAAATAAAATTATGCACAACTCGTACA--TTCACTG

TTTTTATCTACGGATTTTCTGTAAATCACAAATAAACGACTATCCATAGATTTGTGGTAC

GTGCAGGTGCTGTAGTCCAAGGTATATTTATTGCAAGTGTGAAGTCTATGCGAGAATTA-

CAGCCAAAGATATGCGCGTTTTATTTCAGATGTGTGCAAACTCCTATACAAAACGTATGA

GCTACTCG--AGGAGGTGGTTATATACAAGGAATACAAATTCGAATAAAATGTTAAACAA

TATTTAAAAAAAGTAAAATGTTGTTGTTGCTATAAACTAACAGGAAATATGTTTTTTGGA

SCRRTCGGTCCAWGGATGATAAAATAACAGTTAAAACRTTGCTTGAGCATAAGGTATGTA

ACGCTTCTTCGAATTAATGTATAGGGCTTATCCTTACTTTTCCTGACTTTTGCAGTTCTT

TTCCGTAGATATATGACGAGCGTCCAGATAAATATAAGTATACGTGTTATCCGCAAATAA

CCCCTC-TACATSTATTACAAATCGATTAGACCAGTTCGGTAATTATAAACAACAAACAA

ACACAACACCATTGAATGAACTCTCCTCAAGAAGAAGTAC----TTTCGG---CATCAGA

CGACGCTGGATTACGACCGA--TAGCGGTAGTTTAATTAACATATTA-TTAGT--TAAGG

CTTTGGCGTGCCTTCAAAGAGTTGTGTTTTTATTAATTTAAATACTT-TTTGTCT-CGAA

TTTCTATGCTCAGTCTTT------TTCTCGTCAGGCACACTAG---GCAATGATTTAATA

CCGA----TTAAAGTGCAGTATATGTTTGTGTAAGCATCAATACTACAGTCATAT-TGCT

ATGTTCCGCAAAGTTTTTGATGTATGTTTGCCAT-TTGGATGCGATAATAGACAATCAT-

--------AASAAGTTTTATTCTTATCGAATGACGGTATTGCACAATGTTATACAGACAG

TAAGATTTGCGAGTGGKAAATTCCCAATAACAACAGGACAGCTCCACTGATAGGGATGCA

AACCACCCGTGATTCAGTTCAATCGTAGACAGCAGCCGAAATAGACGCAAATTCGCACAA

AGTCAACACCAACTATGCGGCAGTTTATAAGGTCAAGAATATTAACGGTGATAATCTTCA

TAGCAGCCGCACATGGGTAGGAATAAAGGAGC--AAATTTTTGAGTAAGCAA--------

-----TAATTTGGAACCTACGCTTTG-----------TTTCAGGCTACTGGTCATGGGTC

CAAAATGTATYCGGGACAATCAGAACTACACACTTACGATCAGCAACTTTTATTCAAATC

CAAGCAAGTTGGACTTGATGGTGAAGCTGGAAGGGAAAAATGATAATAGTTTAAGCGTTC

TGAACGTGACGAAGATGATCGACGTGCCATGTAAATCTATTCGAATGATCAACTTCAGTG

TAAGAAG----AGTGGTGAAATGTTATAAGGTTTACAAATAAC----AGATTTGCGCTTT

TGCAGATTCCGGATARTCTGTCCTCTGGGAACTACAAAATTACCATTGATGGGCTGCAAG

GTTTCAACTTTCACGAGGAGGCAGAGCTGGTGTATCTCAGCAAATCTATATCRGGAGTGA

TACAAATCGATAAGCCCGTATTTAAACCTGGCGAAACGGTGATATTTCGTGTGATCGTGC

TGGACACAGCGCTGAAGCCTCCGGTGAGGGTCAAGTTCGTTAATGTGATCATTCGGGATC

CTAATGACAATGTGATTCGTCGATGGCCAGCGGCAGAATTATACGCCGGAGTGTTTGAGA

GCAATCTGCAGATAGCRCTCACATCTCTGCTYGGAGTGTGGAACATCWCGGTGCAGGTGG

AAGGAGCAGAGATTGCATCGAAGACGTTTGAAGTGAAGAAAGATGTGTTTTCATGGTTCG

ACGTGCACATCAAGCCATCAGTGATTCCATTAGAAGAGCATCAAGCTCTAAATCTCACGA

TTGAAGCGAAGTATCCCTTTGGCAAGCCAGTGCAAGGAGTGGCCAAGGTGGAGCTATACT

TGGAAGGCGATAGGTTAGATTTGAGTAAAGATRTCAATGTRAACGGGTSAGAACAGGTGA

TGTTWGCKTTTAATGGTGGTTTGGAGTTGAACRACGAACAGCAGGATGTGCGTGTGAAGG

TGGTGRTTGTYGAACAGGATACAGGTGAGTAAAAGGTACCGGGAGAATGTAATGYTTATC

TAGAAAAATATAAWACCAYTTTTTTACAGATCGAACTGTAATGAARGAACAGTCYATCTC

GGTTCACAAATATCCGTATCGTTTGACGATAGTAAAGGATACCTTRCAATTCCGTCCGGG

ACATCATTTCAATTGCACTGTACTGTTGACCTATCACGATGGTAGTCCAGCTAGAAATGT

TTCGCTTTTGGTAGAAGCGACTGGAGAAGGTGTAGAGCACGAGCAGACTTATYTTACACA

GCATGACGGTTCAATTAACGTTTTGTTGCATCCAACACTGTCGACCCAGGAAGTTTATCT

TACTGTAAGTTGAGTCATATATTAMAGATAACATTTTATTAATTATTCCTTTTGGAAATA

TGTTTTTTTAAGATTTY---AGAGAAAAAGTACGATTTATTATACATGAAAAAACTACAT

ACCGTTCAACCTCTTACTAACATCACCATGAAGCTACAACTACGGTTTCCGTAAGTATCG

AGTCG-TGTAGTTGTTTACAGACATTTT----TAAAAGACTATTAA--TTTTTCCAGCAT

AAAACAAAACGAACCTATCATGCTCTTGGCAACGTGCAGCGAGCGGATGACATTTCTCGC

CTACTACGTCGTGTCCAAAAGTAAAATTGTTGATRCGGGTTACATCGGCCAGTTCCGACA

GAAGAAATTTGTGTTCGAAATTAGTGCAACGGAAAAGATGCTCCCGAAGGCAACAATTTA

TGTGACCAGCGCATTCGAAAACATTTTAATTTGGGATGCATTGGAGATAGATTTGAAKCC

GTTGAGCAACGATGTAACTTTATGATTTGTTTGTCAACTCAATGGTTTCCATGGATTAAT

CTTAACTATGTAACTACATTTTCAGCTGGAAATAAGAGTAAACAAATCGAAAGCTAAACC

AGGACAGAAAATAGAGCTGGAGCTGAAAGGACGGCCTAGTGCGTACGTTGGCCTTGCCGC

ATATGATAAGGGTTTGCTGGCGTTCTCCAACCAGCAGGACCCGTTTTGGGAAGATGTTAC

GCAGTTGTTTGACTACTCGCACGAAGACGATCAGACCGAGCTTGATGGATTTCATGTATG

CCGTGTGTTAAATT-------GTTCGATTTTATTTGTATTTCTT----ACGGTGTTTG--

----TGTTGTTCTAGGGTGAAGGAGTATTCCTAAAAATTTCGGGTGGTTTTCAACTAGGA

AACACACTCAAAGTAACTAAACGCACTGTTAAAGACGTTGCGCCTCCACTGGTATATCAA

GCATCTTTGGAAAGGAATTGCATGAAATCCTGGCTGTGGAAAAATGTAATTATTGGAAGC

TCTGGAACCCTCAAACTGGCCGACGTTTTTCCTGAAACGATCACCTCCTGGACCTTGAYG

GGCTTCTCGATTGATCCGATATACGGATTGAGCATCATCAGGAAGCCAATCGAGCTCACA

ACGGTACAGCCGTTCCTCATCGTAGACTACCTACCGTATTCGGTCAAGCGGAGAAAGGAG

GTCATGTTACAGTTTGCACTGTTTAACAACTTTGAACAGGATCACACTGTTGATGTGACG

CTGTATAATATGAACAATGATATGGAAATTCTTGGACGCCCAGTAACCGGTGAGTGTCAA

AGATGGTAATGACGATCCTCAGCWTT---ACTATACAACGAATCTTTGTTTTAGACCAAA

GCTACACCAAAACGGTGAGCGTTCCTCCGAAGGTGTTATTTCCTGTCTCGTTCCTTGTAA

AGGCACAAAAGCTCGGCGAGATGGCGGTCCATGTGACAGCCTCCATAGCCAATGGGCTAG

AGACGGACAYCCTGGAAAAGGTGATACGGGTGATGCCTGAAAGCTTGGTGCAGCCGAGAA

TGGATAGACGCTTTTTCTACTTCAACGATTACAAAAATCTAACGTTTTTGATGTACTTGG

ACATCATAAAGAAGGCTGATAATGGATCGATAAAGATTGAGTTTCGACTAAACCGTGAGT

AGAGGGTTTACAAACTAGTTAAAGACATTAGTAGCAGTTCTTCTTTA------ACCTAAT

CATTTCTGCAGCCAATTTGCTGACCGTGGTCATAAAGAACCTGGGCCATCTTCTTGCCTT

ATCGGCGGGAACTGGTGAGGACAATATGGTCAAATTTGTTCCCAACGTTTTGGTGCTGGA

TTATTTGCATGCCATCGGGTCAAAAGAACAGCATCTTATCGACAAAGCTAYSAATMTGGT

GCGTCAAGGATATCAGAACCAGATGCGCTACTGTCAGAAGGATGGTTCGTTTGGTTTGTG

GGAAAATACAGGTGGTAGCACATTTCTCACCACATTCGTTGCCACAACGATGCAAACTGC

TTCAAAATACATAGTCGAGATAGATGCAGAAATAGTGGATAGGGCATTGGATTGGTTAGC

CTCAAAACAACACAGCTCGGGACGGWTTGATGAGGTCGGTGCAGTGTTTCATAAAGACAT

GAAAGGAGGGTTGCGCGATGGTGTGGCCCTCACATCGTTCGTTTTAACGGCATTGCTGGA

GAATAACATCGCCAAAGTGAAGTACGCAGAGGCGATCCAAAAAGGAATGAGCTATCTGAG

CAATCAGTTTGGATCCATCAACAATGCATACGACCTATCGATAGCAACCTACGCGATGAT

GCTGAACGGACACATCATGAAGGAGGAGTCACTCAATAAACTGATTAATATGTCTTTCAT

TAATGCTGATAAAAACGAACGGTTCTGGAACACAACGAATCCAATAGAAACCACCGCATA

TGCTCTGCTGTCGTTTGTGATGGCCGAGAAGTACACAGACGGTATACCGGTCATGAATTG

GTTGGTGAATCAACGTTACGTTACCGGTAGCTTTCCGAGCACGCAAGACACGTTTGTGGG

CCTGAAAGCGCTGACCAAGATGGCAGAAAAGATATCTCCGTCCCGAAACGATTACACCGT

TCAGCTGAAGTATGGAAAGGATTCGAGAATCTTCAGAATCAACTCAGAGCACATTGATGT

AATGAATTACGTGGRTATACCGGAGGATACAAGGAAAATCAATATCAATGTGAGGGGTGT

TGGGTTTGGGCTGCTGGAGGTGATTTATCAATACGATTTGAATCTCGTCAAATTTGAGAA

TCGATTCAAACTAGATCTGGAGAAACAGAACATAGGCTCTG---ACAAGATRATACTGAA

TGTGTGTGCCAGTTTCATTCCTATGTATCTTCAAAGTCAATCGAACATGGCACTGATCGA

AGTGACGTTACCGAGCGGTTATGTGGTTGATCGCAATCCGATCAGTGAGCAAACGACGGT

GAATCCGATAAAGGTATAACTA--TGTATGTTGTGAATAGTGTATTRATCGCTATTGAAA

ATGTTTAATTCCTT------------ACAGAAAATCGAGACTCGTTACGGCGGCACGTCG

GTTGTTGTTTATTATTATAATATGGACGACAAAAAGCACTGTTTTACTGTATCGGCACAC

AGACAGCAGATGCCGWCATTGAGACGCCCTGCGTATGTGGTTGTGTACGACTATTATGAT

ACAAGTAGGTTATCAGTGTTGATTGACTGTAGAGT-ATATAG---------------TTA

ATTATTATTATCTTT--ATCTTTTTGTAGATMTSAATGCGATAAAAATGTACCASTTAGA

GGACCTGAGTGCTTGCGAAATCAGTGGCGAARTTGACTGTTCCGAGGTGTGCAAAACAAA

GGGACAGGTG--TAAAATAAGMAATTCCTTTC---TTCTATCAATGTGTATGTTCCTTCC

CGTTCGTTGCAAAAACGCACTCCACTGTCCCTTCCCAGACGACATACGCAATTCGGAATC

ACCGATC--TGYTATAAGTCTCCATTTKDAAATTTAACGGATACTCKAGAACTTATTTGA

CTTCTGCATTTCGTCAGAGTCATTTACATTAAAACTCCGATCACTGSAWGKTGGAGCTCA

CTTTTTYCTTCYTCTCTWCATTTATTTTCCTTTTCCTCACTTTCWCACGAATGAAATGAA

ATGAAATAAAGTCTGGGAATACTGATGACAMCGTTACTAGCATACTGAACACAATTATGG

GTTGTCGTCTACTTCATGACGTGGACGGTGACAAACTTACCATTTACAAACTTATCTCAG

TAAAGAAAGGTCGAAAAAACCAAATATACTCACCACGAGCAGCATAATAACATCCATAAC

CGTGG-TGAAAATGAATTTGTTTACAATCGAGAAATCATCTGATGCAAAATATCCTCCCG

CTTTGCCTGCGCATGCGTATCA----AACGTTGGGTTATTTGGTTCCGCTTCGGAAGAGC

GCAAGGA--TCCGTCGTTGACAGTGGAAAATAACCTTTCCGACTGTTGTTGTTGTCTTAT

TCTTTCACCGCATACATGTTGTCATGAAACTGTCAAAACGTGGAAATAAAAAT-AAAGGC

GTGCACGCTCAATGTG----AGATTGGAAAATTGTGTGTG--TTTATTTGGAATCAGTCA

CCATTTCTACACCTAACAAGGTTTTGTGATAAAGA-AGCTTGTTTAGCTGTGCAATTTAT

GCTGTTTGCGGTCGTTTTAA-------CAAACAAATTTACCAAAAAATGTGTGTAAGGAG

TTGTACAACATCGTAAACACAACAGTGCATTAGTTGGATAGTGTGTTGGCTCCAAGCTCC

AAGGTCCTTCGAGCGGACCTTCAAACTCATTAGAAGTGACCTCAAGCATCTCTGATTCGC

TTCCTCCGACACGGATTTGTATGTGTTTCGCAGCCAGTGTGGCTCTGCCTGTGCTACGTC

CAACCAAACACCAGAAGGT-ATCTGCCCAGCGAGTGCATCATAACTCGATCGATAAATTG

ATTTTCCCTTTCTTATCGTTGACGTACGGTGAACGTCGCCGCCGCTGTGATTACATGCAG

CGGCAATACGGCGTGCTTCAAAAATAGATGATTCCATCAAGAAAAAGCAA---TATTGTC

CTGCAACATTAATGTGA---CCGCAGATTGTTTGAGAAGC-AGTTGCAGCTGGCGTGCGA

ACGGTATGTTGGCCTTCGTTGGTAGCCGTTCGTACTGTGTATACACAGTGTGAGATTGTT

GGCTCGTACGAAAAGTTGTGCGGTTTCGTGCCTCAAGTGAGACAGTGCAGTTTGGGGTGG

GCTAATGAACGAGGGAAGTGTGCTGAGATACGCAAATACGA--CAGCACGTGCAAGACAA

AGGACGTCTGTTTCATCGAGAAGATCAAACGAAACAAAC--CAGCATTCCGACCGTGCGA

CATCGAGTGATGATCGTTTTGTTCACT-GTTGTGCCGCTAGTTGTAGATCACCAATCATA

ATCAATGTTTCAGGTAAGTTGCTRAMATAGRAAACGGGAGGATTWTAATWGTTTTTATTC

ATAGTTTCYAGGGYGGTGRRAGGRGGGCKGGCTTCATTGTATTWCARACAATCAATGAAC

TTACAATYTYYGTGAT----TTGTAACAAGCTTTAGATCAAAACAAGWAYCAGACAAGTA

AAAWMTATARCTAATAATCTAACAATTAACCCAATWMGATCAATTACACCGTTTTCGAGC

CCAAATAAACAATGTTAAGCTGCTTTTAAATTTTGTTCAATTATAAGTAAACGTTTGATC

GCCAAC-TGAAATTCTGATGCTTTTCGGAACTTCTTTCAATCGCAACTAAACAAGTGATA

CCCACGACTTGATCCGTTTGCTGTATGGAGTTCAGAGTTTTATTTGTGCCTTCATTT---

------ACAAAAATAATTTGAATGTATTTGCCAATAATGTATCC-----CGATAGTTTCA

AAGTTAATAATTGACTATTCAATCAAAATCAATTAAAATGAGGCTCCAAACAATTTAATT

GCAACAAAATGTTCATCGAAGAAACGAACCTGTGTAAAATAAATATACGTTAATATTATT

GAAATCA-----AAAGTTTAGATGCAATTGAACGAAGTTCAGAAAAGCATCAGAATTTCA

GTTGGTCGTCAAACTTT--TACTCATAGCTAAACAATGTTTAAAAGCAGCTTAACATTGT

TTGATTGGGCTCATCCTATAATTACCCTGTAATGTAAGATTCGCTAGTATGACGATCGTT

TTTTTTTTGTTTACGGTATTTTACGGTATTCAACGTATTTAACTTGATGTATCAATTTGT

TTTCAGTTTTATGTTTTGTTGTGTGACCAAAACCAAAACGACGAAATTGAATAACCCAAT

AGTGCTTGTTTGTATGATAAAGTTAAAGTTTGTTCAGTTTGTTCAAAGTTTGCAAGTTGC

TGCACAGTTATAACAATTTGATATTTTCTTATATTAACCTATTCTATTAAACA-ATCAAG

AAAGCAGAAATTCTGCCAGTGGAACTATACTCATCATCAAGGTTGATCCATTTGACTTAT

CTGTCA--TAGTGGATTTTACCATTTGTGCACAAAAAATACATTTAAAATCGATTTGTTT

TCYTTTTTTCTGTYTGRAATGTKTT----CTAGTGCTTTGAAGAAATTKTRAAATACCGA

STAAAACAAWTAATAACCTTTMTTGGCACAATGTTTATGCATTAGCCAAAAACAAACCAA

ACACATGCATGAGCCTCGAACCGGTAAGRCAGCCACGWATCGCTGTTTCTTTGCATATTG

GTTTTCAAG-AAGAAATAATTCACCCTGAAAATCTGAGGCTCRTTAGGCGTGTGATTTTG

TGTAGRGTGTTGACRTGAAAACTTCATATAAAAATTGGCTAGAATCATAAAACCCCTCAT

CGATTTATTTCAGCAAYATTTTGTAGCTTTWTGATTCCAGCTAGAATGTWCGATGAAGTG

TGACATGTTGTAGCATAAAATAWTTTCA-RMAGTTTATTGGATTTTWAAAAGATCTCTCA

CTSTTTTCGACCAGGAGAACAWGTTTTRAATTCCAATATGTCAGATTGATCGTCCCGTGC

CGTTGTCAACTCGCTTCACTTGTATCCTGCTACGGGTAATCAATAAGTCTCTGATAGCCA

AGCCCCATTAGTGGTACAGGCAGACCTTGAACCGCAACCGTTATTGTTCCACAGAAGAAA

AATATGTGAGATGCTTCATTGGTATGGACTTGATTTAC-ATATTAGGAATTCAACCAATA

GAAGTAAGCTTGATTCTCACAAGCTGATGATTAAAATTCTCTAATTTATGTCAGCTCCCT

TTGGTGATAAAAGGTGCAAGGAAACTTGCTTGTCAGACCATATGTCCCTGAAAGGCCCGC

TGA-TAATGTTCCCAGAAGTAAGCCGGAGTCTTACGATACAACTCCGCAGATCCCGGTAT

CGTCAACGTTTTCACGTATCGTTCCGCAGTCTTACAATTGACTCATGACGATTCATCAAA

GTGCCCCGCGGTCTTGAGCTTCATCGCAAAACCCAGTAGTTATATTAGTAATTTTAAAAA

AATCCCACTTTTTTCAGTGAATTGAGCATG-----CTGTTAAGTCGTTCGAGCTATCGAC

TGTACCACGAAACCGGCTTGAAGA-CAATATACTTTAAAAAACATCAAATCTACTTTGGT

TTCACTATTCCTACCTTGTTTCATGAGCAGCATAAAGCTCTAATAACGATGTATACAACT

TGCTTAAAAATGAACCCACATCTTGATGATTCTATTTTAATAAACCAAGATGTATCGTCG

GCCCCAGTTAAGACGTCAACTAACAGTCAAAAATACATCTTCCCTGCCTTTCGAGGGCTA

GGGGGAACCGGAACGCTGA-----------------------------------------

------------------------------------------------------------

--------------------------------

TEP5 (Ensembl TEP 17)_region from traces

------------------------------------------------------------

------------------------------------------------------------

------------------------------------------------------------

------------------------------------------------------------

------------------------------------------------------------

------------------------------------------------------------

------------------------------------------------------------

------------------------------------------------------------

------------------------------------------------------------

------------------------------------------------------------

------------------------------------------------------------

------------------------------------------------------------

------------------------------------------------------------

------------------------------------------------------------

------------------------------------------------------------

------------------------------------------------------------

------------------------------------------------------------

------------------------------------------------------------

------------------------------------------------------------

------------------------------------------------------------

------------------------------------------------------------

------------------------------------------------------------

------------------------------------------------------------

------------------------------------------------------------

------------------------------------------------------------

------------------------------------------------------------

------------------------------------------------------------

------------------------------------------------------------

------------------------------------------------------------

------------------------------------------------------------

------------------------------------------------------------

------GCGGCACAGAACCATACAAAGAACCATAAATTACCTATGTGCCAGATTTGGATC

AAATCAACGT------TGTTGTTATTGTGTGATTAACAACTGTTCATA--CA-GATGGTA

ATAGAAACGC----TCG---------TTTTTAGTTATGAGA------TAAAACAATAATT

AAAAAAAATGTTAATTTAGAAGATTCAATAAAAAATAAATTTAATTTGGTCACGTGTATT

GTAGCATGAAGAGCTGACTTCAT--CGTCGTGCTAAAGTCAAGCGACAATTACCACTAAA

GCATTTTAAAAGTTCAATGTTATCATTTCTTCGAATGGAAAAGCCCTCAATTCAAAAATA

TTTTTGTAGCATTTCCGTGATAACAGTTTCATATGATTGCCTGGGTTTTCCCAATGACGG

TTTACGTTACCCCAAATAT-ATTCCATAAAATGGAATTCC---CCAA---AGTT-TTTGT

TTCACTTTATT--CTTCTTCACTATCGGAAATCGGCGCAACTTTGCTATAATCACACAAC

GCCACATAAAA-ACCATGTGGCTGTTC--ATAAGGTCACGAATATTAATGGTGATAACTT

CCATAG-GCGCTGAACATTGATGGGTGCAAACATGCTGGAAGTGTTGTGTA-CTTCGAGG

GAGATGAAAATAGATGCACTGCACATTTCAAC----TTCCTT------TATCTAGGC---

-----TATGACATAGTAT-------AATACTGAAATGTACCCGGGCCAACGAGGA-----

---CTACACACTGGCAACTTTAACTT---ACACTT---AACCAAAGGG-GAGCTTTATAG

TGTAGTTAGAATTTTCTGAACGGGACAAAAATGGTTGAAAAGCAACAGAAT----ATCAA

CCGGATGAGAAATGTCAATGTAGGAAGCAATCGTTACGAATGCTAGCAACAAAAAATGGA

AAAAATATGGGGTCCA----GGATGTTACTCCAATGCATTCATAAACAATCCAGAAAACC

AAGAAACAATCAATCAGCGCGTTGAAGCATCCGGAAGTACATTGTACCTTCGTTCCGCTT

AC---TTTATATTTTGACAGTTTGGTGGTTAGCAGAGAGCAATTCACAGAAACAAAGGCA

GTGCA--GATACGGGTTAGAAAGTATGTAGGTGTATACTGTTYCAAGAGATTTTTCAAAT

TTA-AAGATGTGCG-ATGTGTAGTACAATAAAACTCTTTTAAGTCCGCGTGGTCGTTACA

TCTAATTACATTAGGA--AATGGATCGATAACCTGGACGAAAAAAAAAATAGTCAAAAGA

GGATCACATGGTGTTTTGAGATCATTTTTGTATCATTTCCCTTGCTTTTTTATCTATAAA

CATAATAACCTTATATACGCACGGTGCTTATCACGCCAACTTGTACATGGTATTTTTGTA

GCAAATAATCAGCCCTCTGCTATTGTTGGT-TTAATACCAGTAGAACTGCAGTATTTGAT

TGTGTAAGCAACAATACTATAATCACGCTCCAATATT------CAGACAAGGTATTTTT-

------TGGATGTGATTT--TAAAGAAGCATAGGCTTTATTCTTATCAAACGGCATGAGT

TAATATTTGTGAACGGGAAATCCCAAAAAACAGCACCACAGCACCACCTATAGGGATGCA

AACCACCCGTGTTTCAGTTTCTTGGTGGACAGCGTTCAAACCATACAAAAAATTGCACAA

TGTCTAAATCAATCATGTGGAAGTGTATTAGGTCACGAATATTAATGGTGATAATCGTCA

TAGGTGCCGCTCAAGGGTAGGAACAATCGTGCTGGAAGTGCTGTGCAAGTAGATGCA---

---------TTGGAAACTGCAATTTGTCAT-TTCATTTTCTAGAGTACTAGTCGTGGGTC

CGAAATATATCCGAGCCAACCAGGAATACACTCTGGTGATCAGCAACATTAACTCAAACC

AAACCAAATTGGACTTGATAGTGAAGCTAGAAGGCGAAACTGATAATGGTTTAAATGTGT

TGAACGTTACCAAGACTTTCGACGTGCGACGTAATATGAACCGAATGATCAATTTCAATG

TAAGAAGTGACAATACTGTTTGGTTATAAGTAGCAAACCTTAAGCCAACCTTCGATCCTT

TGCAGATGCCCGAGAATCTTGCTGAAGGGAACTACAAAATTACCATCGATGGACAGCAAG

GCTTCAGCTTTCACAAGGAGGCAGCGCTGGTGTATCTCAGCAAATCGATATCGGGGYTAA

TACAGGTCGATAAGCCCGTATTTAAACCTGGCGATACGGTGAACTTCCGTGTGATCGTGC

TGGACACGGAGCTGAAGCCGCCAGCGAGGGTCAAGTTGGTTTATGTAACTATACGAGATC

CTCAGCGCAATGTGATTCGCAAATGGTCCTCGGCAAAACTGTATACCGGTGTGTTCGAGG

GCTATCTGCAGATAGCGCCTACTGCAATGCTTGGAATCTGGAATATCTTGGTGCAGGTGG

AGGGAGAAGAGCTTGTGTCAAAGACGTTTGAGGTGAAGGAGTATGTGTTGTCAACGTTCG

ACGTGCAGGTCATGCCATCGGTGATTCCACTGGAAGAGCACCAAGCTGTGAATCTTACAA

TCGAAGCGTACTATCACTTTGGTAAGCCAGTGCAGGGAGTGGCCAAGGTGGAGCTTTATC

TAGACGATGAGTTGATAGATCAGAAGAAAGAACTGACTGTGTATGGAAAGGGTCAAGTAG

AGTTGCTGTTTTTTGGTAAATTTGAAATGTACGAAGATCAGCAGGATGTACGAGTGAAGG

TGACGTTCATCGAGCAGTACACAAGTAAGTTTCCAGCTC---ATTACCRCTTAGAAAACT

GTTAGTGATGTCTAATATTCTTTTCACAGATCGTACGCTGGTGAAGCTGTCACAAATAAC

GATATACAAGCATGCGTACCGAGTAGAGTTGATCAAAGAGAGCCCTCATTTTCACCCGGG

AATTCCGTTCAAGTTTGTGCTTCAGTTTACACACCATGATGGAACACCGGCTAAAGGCAT

TACCGGGAAGGTAGAGGTATCCGGTATAGGATTCTAA-ACGA-CAGCAACGAGTGATA--

--ACGATGGATTGATTAAGCTCGAGCTGCAAAAAAATGAGGATATCGAATCAATGCACGT

TAGCGTAAGT-GTTTC-AATTTT-----TGTTGATTTAAGGAATTATAATTATAAATCTT

TACTCATTTCGCAGTTTGTAAATAATAATGATGGATTTGTCTTTGAACACAATGTTG-AC

AGAGATGTATTCGCTACAAATGCTTACATTAAAATAGAGCTGAAATCACCGTGAGTAACG

ACCAGCTACAAAGTGAAACAGGCAGTGTAATGTACAGTATAACCAAACTTGTTCTAGCAT

CAAACTGAACAAAGTGATGCGCTTCACGGTGACGTGCACGGAGCGCATGACATTCTTCGT

GTACTATGTTGTGTCAAAGGGCAACATCGTCGATGCAGGCTTTATGAGACCAAACAAGCA

AAAGAAGTACTCGTTGCAACTAGACGCTACTGAAAAGATGATTCCGAAGGCGAAAATCCT

TGTCGCTACCCTAGTGAATCGTACGATGGTGAACGATATTGTGGATATTGATTTCCAAGG

GTTTCGTAATAACGTAAGT-----GGCCATTTTTTTAAGTATGTTGTTTGAAGAATTA--

CTTATTCGTGTTG-TACGAAAACAGTTTGATTTAAGCATTGACGAGCAAGAGATCAAGCC

GGGACGTCAAATCGAGCTGAGCATGTCTGGACGCCCTGGAGCGTACGTTGGGCTGGCCGC

GTATGACAAAGCCTTGCTGCTGTTCAACAAGAACCACGACCTGTTCTGGGAGGACATTGG

GCAGGTGTTTGACGGGTTCCATTCATATGACACAAATGAATTCGACCTGTTTCACGTAAA

TAAAAGATTTTAGCATAGATACCTTAGCGAAATTTTTAATTTTTAAAAATTTGCTTCGAT

TGATTCGTATTTTAGAACATGGGCTTGTTTGCCAGAA---TGGATAATATTATGTTTGAT

CAATCCAATGATAAATCGGCACGCAGTGGACAGCAGGCGGACGGAACAGT----------

-----GTTTAGGAAACAGTTTCTAGAATCATGGTTGTGGAAGACTGCGATCATTGGAAAT

TCCGGTACTCTCAAGCTGATTGAGGTTGTACCGGATACAACGACCACCTGGTATCTGACG

GGCTTCTCGATCGATCCCGTGTACGGGTTGGGTATCATTAAGAAACCGATCGAGATCACA

ACAGTGCAGCCGTTTGTCGTTATGGAAAGCTTGCCGTATTCGATCAAGAGAGGTGAGGCG

ATCGAGATACAATTTATTCTTATCAGCAACCTTCAAGAAGAGCACACCGTTGACGTGACG

CTGTATAATGAGAACAACGAGATGGAGTTCATCGGACGTTCGATTTCAAGTAGGTATCAA

AGGGAATAATGACAATATAATACTT---AGCTTCACC-----TTTTTATTCTAGACGTAA

GCTACACCAAATCGGTGAGTGTACCTCCAAAGGTAGGAAAACCTGTATCATTTCTAGTGA

AAGCAAAGAAGCTCGGTGAGATGATGGTTCGTGTGAAAGCATCTATAGCCAATGAGCTTG

CAACGGATGCACTGGAGAAGGTGATACGGGTCACGCCAGAAAGCTTGGTACAGTCAGGAG

TAGAATCGTTCGGATTTTTCATGGACACGCATCAGAATCGAACGTTCTTGGTGAATCCGA

ACATTGATAAAAAGGCCGATAATGGATCGATAGCGATCGAGCTTAGAGTTAATCGTGAGT

ATTCAAAAAAAGAGTT-TTGCTCCCAACACATTAAAATCAAATTGT------------AT

CGTCCCCGCAGCCAACTTACTGATTAAGGTCAAAGAAAATTTAAATGATCTTCGTACTGT

ATCGCCTACATGTA------AGAATGTGATAAGATTGGCACTTAATTTTGCTGTCGCTGA

TTATCTGATTGCATGTGGACCCAAAGAACAGAATCTTTCCGAAAATGCTGTCGATGCGTT

GAGTAAAGAATATATGTTGCTAATGACTTGCTTGAATTCGGATAGTTCATTTGATAGCGT

GCAGAATATTGTTAGCAATATATTTTACACTGCATTTGTAGCCAATACGCTGGATACTGC

ACCCAAGAATGTGCGCCAGACAAGTAATGTTAAATTAGAGAAAGCATTTGACTGGCTTGC

CTCTCGACAGCAACGTTTCGGAAGTTTCAGAGAAATCGAATCCGATCTGCACTA-----T

ACA----------CGTAGTGAAATAGCATTGACATCCTACGTATTGGCAGCAATGCTAGA

GAACGAAAGCGCCAAAGTGAAGCACGCTGTGGTGATAGAAAAGGGCATGTGGTTTCTGAG

CAATCAATTCGATTTAATCACAAGYGCAAATGATTTGGCGATAGTGACCTATGCGATGAT

GTTGTACGGGCACTGGCGAAAGGACGCTGCGTTCGAAAAGCTGATCGATATGTCAACCAT

CACAAATAATGGAGCGGAACGATATTGGAACACGTCGAACAGCGTAGAGGCAACGTCTTA

CGCTTTGCTATCGCATGTGTTGGTCAACAAGTTGCTAGCAGCTTTGCCAATAATGCGTTG

GCTAGTGAATCAGAAAAGCGAAATAGACAGTGTTTCTGGCCAAGAAACTACCTATTTGCG

TCTCAAAGCACTGTCCAGGATGACAAAAAAGATATCTCCATCCCGGAATGACTATATGGT

ACAGCTAAAGTACAAGCAAAGTACGCGATTGTTGCGTTTTGATTCATACAGAAATATGAT

ACAAAACATTAC---CACACCGCAAGACGTAAGGAAGATTAAAATAACAGTTGAGGGTAT

TGGGGTTGGGCTCCTGRAGGTGATGTATAAATACAGATTGAATCTCGTGAACTTTGAGCA

TCGATTCCAGCTAGACTTACAGAAACAGAACACAAGCTCCGACAACGAGCTAAGGCTGAA

TGTGTGTGCCAACTATATCCCCACTTTGCGTGACAGCCACTCAAACATGACACTAATCGA

GGTGACATTACCGAGCGGTTACGTTGTCGATCGCAATCCGATCAGCGAGCAGACGACGGT

GAATCCGATACAGGTAAGCATATTTGAATGCTGTAAATGGAGGATACTTGGAG----CTA

CCATTTAATTGCTTGGAATTTYATTCACAGAACATGGAAATTCGTTACGGTGGCACTTCA

GTCGTTCTGTACTACTACAATATGGGCACCGAGCGTAACTGTTTTACCGTGACTGCGTAC

AGACGCTTTAAGGTCGCATTGAAGCGTCCAGCGTATGTTGTTGTGTACGATTATTATGAT

ACAAGTGAGTAGTAGTCATAGATTGGCTTTGGAATTGCATAGGGATTGGCTATGGAATTG

ACACTTGTTGCTTTTTAAATTGTTTACAGATCAGAACGCCATCAAAGTGTACGAAATGGA

CAAGCAGAATGTGTGCGAAATCTGCGACGGTTGCGGCTGTACCGCAGAGTGCAAGACATA

AT--TTGAGC------------ATTTTTTTTCGGTTGTTATGAATG-----TTGA--TAT

TTTTATGTGTTAAAATTCG-TATTTTTCTTGTCATAGGTTGACACAATTATTRTA-TGTT

TA--AGCGAAGAAAAAAATATTGAAATTGT-ATTAAYAATATATTTGAAAAGACATCTTA

GTA----------TGTGAAACTTTTA--GGAAAACTT-------TG-AACGAACAAAAAC

----TTTTTCCAGTAGTAAAAAGGCAAACTTCTATATCGCGGGGTGTTGGGTGGTTAAAG

ATG---TACAAATTGGGATACAAGTTCAATCTACACTACGTTAAATAAGAGTTGAAATAA

AATA---AAATTCTGAGATTTTCAATACTAATTAGCAAATACAG--CG--CCTATCACAA

CTAT------TTGGATAGTCGTTTTTTGCGATTTTCGGTAA---------AATCGTAAAT

TATAGATAAAG---ACAGTGAATGTATGAGTTGTGCAAAATATTATATCACATC-TATCA

CGTTAATCATAAAAAAATTTGA--ACATTTTTGAACGACTTATTA-------CGAAAAAA

AAAACAA--TTTA--ATGGTCGTACCATAATTATAAAAACACTTCGAAA-AACAAGTTAT

TTGT--ACTAACCTAAAAT---CGTTGAATCATACGCTGATG-ATATAGACATCATTGGT

-CTGCGGCTCTCCTAT----GTAGCAGAAGCCTACCAAGGGATTGAG-------------

------CAGGCGGCAGAGAACCTCGG-ATTGCAGATAAAC------GAGGCAAAGACCAA

ACTGATGGTGGCAACATCA-----------GCGGACCTACCAATAAAT-AATCCAAATCT

ACGTAGG-CGTGATGTACAGAT-AGGTGAACGCACTTTTGAAGT--CGTCCCAGA-ATTC

ACCTATCTTGGGTCAAAGGTC----AGCA--ACG-ACAACAGTATGGAAGTTGAGTTGC-

GCGCAAGGATGCTGGCTGCCAACCGGTCATTCTACAGCCTGA----AAAAGCAGTTCACC

TCAAAGAACCTGTCGCG--------ACGGACGAAGCTGG-G-ACTATATAG--TACCTAT

ATAGTACC--AGTACTCACATACGCCTCTGAGACATGGACACTGTCCAAATCTGACGAAG

CCCTCTTAG---CCGCGTTCGAGAGGAAGATGCTCAGAAGGATA---------CTTGGCC

CCGTATGTGTGGAAGGA---CAATGGAGGAGCCGCTAT---AATGACGA--GCTATACGA

GATGTACGGCGAC-CTCACTGTCGTACA---GCGTATT--AAGCTC-GC-CAGGCTCCG-

-GTGGGCTGGCCATGTTGTACG-CATGGAAACGGACGACCCAGCCCGTAAAGTCTTTTTA

--GGCCGTCCACAAGGACAGAGGAGGCGTG-GTAGGCCCAAATTGAGGTG-GCAAGATGG

CGTGGAGGCGTCCGCCATT--AAGGCCGGGATAACGGAC--TGGCAGACGAAG-GCGCGA

GACCGTGAGCGG----TTTCGGACACT-CCTGAGGCAGGCC---AAGACCG-CAAAGCGG

TTGT-AGCGCCGGATAAGTAAGTAAGTAA-------------------------------

------------------------------------------------------------

------------------------------------------------------------

------------------------------------------------------------

------------------------------------------------------------

------------------------------------------------------------

------------------------------------------------------------

------------------------------------------------------------

------------------------------------------------------------

------------------------------------------------------------

------------------------------------------------------------

------------------------------------------------------------

------------------------------------------------------------

------------------------------------------------------------

------------------------------------------------------------

------------------------------------------------------------

------------------------------------------------------------

------------------------------------------------------------

------------------------------------------------------------

------------------------------------------------------------

------------------------------------------------------------

------------------------------------------------------------

------------------------------------------------------------

------------------------------------------------------------

------------------------------------------------------------

------------------------------------------------------------

------------------------------------------------------------

------------------------------------------------------------

------------------------------------------------------------

------------------------------------------------------------

------------------------------------------------------------

------------------------------------------------------------

------------------------------------------------------------

------------------------------------------------------------

------------------------------------------------------------

------------------------------------------------------------

------------------------------------------------------------

------------------------------------------------------------

------------------------------------------------------------

------------------------------------------------------------

------------------------------------------------------------

------------------------------------------------------------

--------------------------------

Spliced_TEP1

------------------------------------------------------------

------------------------------------------------------------

------------------------------------------------------------

------------------------------------------------------------

------------------------------------------------------------

------------------------------------------------------------

------------------------------------------------------------

------------------------------------------------------------

------------------------------------------------------------

------------------------------------------------------------

------------------------------------------------------------

------------------------------------------------------------

------------------------------------------------------------

------------------------------------------------------------

------------------------------------------------------------

------------------------------------------------------------

------------------------------------------------------------

------------------------------------------------------------

------------------------------------------------------------

------------------------------------------------------------

------------------------------------------------------------

------------------------------------------------------------

------------------------------------------------------------

------------------------------------------------------------

------------------------------------------------------------

------------------------------------------------------------

------------------------------------------------------------

------------------------------------------------------------

------------------------------------------------------------

------------------------------------------------------------

------------------------------------------------------------

------------------------------------------------------------

------------------------------------------------------------

------------------------------------------------------------

------------------------------------------------------------

------------------------------------------------------------

------------------------------------------------------------

------------------------------------------------------------

------------------------------------------------------------

------------------------------------------------------------

------------------------------------------------------------

------------------------------------------------------------

------------------------------------------------------------

------------------------------------------------------------

------------------------------------------------------------

------------------------------------------------------------

------------------------------------------------------------

------------------------------------------------------------

------------------------------------------------------------

------------------------------------------------------------

------------------------------------------------------------

------------------------------------------------------------

------------------------------------------------------------

------------------------------------------------------------

------------------------------------------------------------

------------------------------------------------------------

------------------------------------------------------------

------------------------------------------------------------

------------------------------------------------------------

-----------------------------CGGCATCCGAAGCAGACGCGAAATCGCACAA

CGTCCAAACCAACCATGTGGCAGTTCATAAGGTCACGAATATTAACGGTGATAATCTTCA

TAGGTGCTGCTCATGGG-------------------------------------------

--------------------------------------------CTACTGGTTGTGGGTC

CGAAATTTATACGGGCCAACCAGGAATACACTCTGGTGATCAGCAACTTTAACTCACAGC

TAAGCAAAGTGGACCTGCTGTTAAAACTGGAAGGCGAAACTGATAATGGTTTAAGCGTTC

TGAACGTTACCAAGATGGTTGACGTGCGACGTAATATGAACCGAATGATCAACTTCAAT-

------------------------------------------------------------

-----ATGCCTGAGGATCTGACGGCTGGAAACTACAAAATAACTATCGATGGACAGCGTG

GCTTCAGCTTTCACAAGGAGGCAGAGCTGGTGTATCTCAGCAAATCGATATCGGGGCTAA

TACAGGTCGATAAGCCCGTATTTAAACCTGGGGATACGGTGAACTTCCGTGTGATCGTGC

TGGACACGGAGCTGAAACCGCCGGCGAGGGTCAAGTCGGTTTATGTAACTATACGAGATC

CTCAGCGCAATGTGATTCGCAAATGGTCCACGGCAAAACTGTATGCCGGTGTGTTCGAGA

GCGATCTACAGATAGCGCCTACTCCAATGCTCGGGGTCTGGAATATCTCGGTGGAGGTGG

AAGGAGAAGAGCTTGTGTCAAAGACGTTTGAGGTGAAGGAGTACGTGTTGTCAACGTTCG

ACGTGCAGGTCATGCCATCGGTGATTCCACTGGAAGAGCATCAAGCTGTGAATCTTACAA

TCGAAGCGAACTATCACTTTGGTAAGCCAGTGCAAGGAGTGGCCAAGGTGGAGCTGTACC

TAGACGACGATAAGCTAAAACTGAAAAAAGAGCTGACTGTGTACGGAAAGGGCCAGGTAG

AGTTGCG---CTTTGACAATTTTGCAATGGATGCGGATCAGCAGGATGTACCAGTGAAGG

TGTCGTTCGTCGAGCAGTACACAA------------------------------------

-----------------------------ATCGTACGGTGGTCAAACAGTCACAAATCAC

GGTATATAGGTATGCGTACCGAGTAGAGTTGATAAAAGAGAGTCCACAGTTTCGTCCGGG

ACTCCCGTTCAAATGTGCGCTTCAGTTTACACACCATGATGGAACACCGGCTAAAGGCAT

TAGCGGTAAGGTAGAGGTATCCGATGTACGATTCGAA-ACGA-CAACAACGAGTGATA--

--ACGATGGATTGATTAAGCTCGAGCTGCAACCAAGTGAGGGTACTGAACAACTCAGTAT

TCAC--------------------------------------------------------

--------------TTC---AATGCTGTTGATGGATTCTTTTTTTATGAAGATGTGA-AT

AAGGTAGAAACGGTTACGGATGCGTATATTAAACTGGAGCTGAAATCACC----------

---------------------------------------------------------CAT

CAAACGGAACAAATTGATGCGTTTCATGGTGACGTGCACGGAGCGCATGACATTCTTCGT

GTACTATGTCATGTCAAAGGGCAATATCATCGATGCAGGATTCATGCGACCCAACAAGCA

ACCGAAGTACCTGTTGCAGCTGAACGCAACAGAAAAGATGATTCCGAGGGCGAAAATTCT

CATCGCTACCGTAGCGGGCCGCACGGTGGTGTACGACTTCGCAGACCTCGATTTCCAAGA

GCTTCGCAATAAT-----------------------------------------------

-------------------------TTTGATTTAAGCATTGACGAGCAAGAGATCAAGCC

GGGACGACAAATCGAGCTGAGCATGTCTGGACGCCCAGGAGCGTACGTTGGGCTGGCCGC

GTATGACAAAGCCTTGCTGCTTTTCAACAAGAACCACGACCTGTTCTGGGAGGACATTGG

GCAGGTGTTTGATGGGTTCCATGCAATCAATGAGAACGAGTTTGACATATTCCAC-----

------------------------------------------------------------

---------------AGCTTGGGTCTGTTCGCCAGGACATTGGACGATATCTTGTTCGAC

AGTGCAAATGAAAAGACGGGGCGTAATGCACTGCAGTCAGGCAAGCCGATCGGCAAGCTG

GTGTCGTATCGGACGAACTTCCAGGAATCGTGGTTGTGGAAAAATGTTTCCATCGGACGA

TCGGGAAGTCGCAAGTTGATCGAGGTAGTACCGGACACGACCACCTCCTGGTATCTGACG

GGCTTCTCGATCGATCCCGTGTACGGGTTGGGTATCATCAAGAAGCCAATCCAGTTCACA

ACAGTCCAGCCGTTCTACATCGTAGAGAACTTACCATATTCAATCAAACGAGGCGAAGCG

GTTGTGTTGCAGTTTACGCTGTTCAACAACCTTGGAGCGGAGTATATAGCCGATGTGACG

CTGTACAATGTGGCCAACCAGACCGAGTTCGTCGGACGTCCAAATACGG-----------

------------------------------------------------------ATCTCA

GCTACACCAAATCCGTGAGCGTTCCTCCAAAAGTTGGTGTGCCAATCTCGTTCCTCATCA

AGGCCCGCAAGCTCGGCGAGATGGCGGTTCGTGTAAAGGCTTCGATAATGCTGGGACACG

AAACGGACGCCCTGGAAAAGGTAATACGGGTGATGCCTGAAAGTTTGGTGCAGCCGAGAA

TGGATACACGCTTTTTCTGCTTCGACGATCACAAAAATCAAACGTTTCCGATCAACTTGG

ACATCAACAAGAAGGCCGACAGTGGATCGACAAAGATTGAGTTTCGACTAAATC------

------------------------------------------------------------

-----------CCAATTTGTTGACCACGGTCATCAAGAACCTGGACCATCTTCTCGGCGT

TCCGACGGGATGTGGTGAGCAGAATATGGTCAAATTTGTTCCCAACATTTTGGTACTGGA

TTATTTGCATGCCATCGGGTCGAAAGAACAGCATCTAATCGACAAAGCTACGAATTTGTT

GCGTCAAGGATATCAAAACCAGATGCGCTACCGTCAGACGGATGGTTCATTTGGTTTGTG

GGAGACTACTAATGGTAGCGTGTTTCTCACCGCGTTCGTTGGCACATCGATGCAAACTGC

AGTAAAATACATAAGCGATATTGATGCAGCAATGGTGGAGAAGGCATTGGATTGGTTAGC

CTCGAAGCAGCATTTCTCGGGACGGTTTGACAAGGCCGGTGCAGAGTATCACAAAGAAAT

GCAAGGAGGGTTGCGCAATGGTGTGGCCCTCACATCATATGTGTTGATGGCATTGCTGGA

GAATGACATTGCCAAAGCAAAGCACGCAGAGGTGATTCAAAAAGGAATGACCTATCTGAG

CAATCAGTTTGGATCCATCAACAATGCATACGACCTATCGATAGCAACCTACGCGATGAT

GTTGAACGGACACACCATGAAGGAGGAGGCACTCAATAAGCTGATTGATATGTCTTTCAT

TGATGCTGATAAAAACGAACGGTTCTGGAACACAACGAATCCAATAGAAACCACCGCATA

TGCTCTGCTGTCGTTTGTGATGGCCGAGAAGTACACAGACGGTATACCGGTCATGAATTG

GTTGGTGAATCAACGTTACGTTACCGGTAGCTTTCCGAGCACGCAAGACACGTTTGTGGG

GCTGAAAGCGCTGACCAAAATGGCGGAAAAGATATCTCCGTCCCGAAACGACTACACCGT

TCAACTGAAGTACAAGAAGAGTGCAAAATACTTCAAAATAAACTCGGAGCAAATTGATGT

GGAAAACTTCGTGGATATACCGGAGGACACAAAAAAGCTCGAGATCAATGTGGGGGGCAT

TGGATTTGGGTTGTTAGAGGTGGTTTATCAATTTAATTTGAATCTCGTCAACTTTGAGAA

TAGATTCCAACTAGACCTGGAGAAACAGAACACAGGCTCTGACTACGAGCTGAGGCTGAA

GGTCTGTGCCAGCTACATACCCCAGCTGACCGACAGACGATCGAACATGGCACTGATTGA

GGTAACCTTACCGAGCGGTTACGTGGTTGATCGCAATCCGATCAGCGAGCAGACGAAGGT

GAATCCGATTCAG-----------------------------------------------

------------------------------AAAACTGAAATCCGTTACGGTGGCACTTCA

GTCGTTTTATACTACGACAATATGGGCAGCGAGCGTAACTGTTTCACCCTGACCGCGTAC

AGACGCTTTAAGGTCGCATTGAAGCGTCCAGCGTATGTGGTTGTGTATGATTATTATAAT

ACAA--------------------------------------------------------

-----------------------------ATCTGAACGCCATCAAAGTGTACGAAGTGGA

CAAGCAGAATTTGTGCGAAATCTGTGACGAAGAAGACTGTCCTGCAGAGTGCAAAAAATA

G-----------------------------------------------------------

------------------------------------------------------------

------------------------------------------------------------

------------------------------------------------------------

------------------------------------------------------------

------------------------------------------------------------

------------------------------------------------------------

------------------------------------------------------------

------------------------------------------------------------

------------------------------------------------------------

------------------------------------------------------------

------------------------------------------------------------

------------------------------------------------------------

------------------------------------------------------------

------------------------------------------------------------

------------------------------------------------------------

------------------------------------------------------------

------------------------------------------------------------

------------------------------------------------------------

------------------------------------------------------------

------------------------------------------------------------

------------------------------------------------------------

------------------------------------------------------------

------------------------------------------------------------

------------------------------------------------------------

------------------------------------------------------------

------------------------------------------------------------

------------------------------------------------------------

------------------------------------------------------------

------------------------------------------------------------

------------------------------------------------------------

------------------------------------------------------------

------------------------------------------------------------

------------------------------------------------------------

------------------------------------------------------------

------------------------------------------------------------

------------------------------------------------------------

------------------------------------------------------------

------------------------------------------------------------

------------------------------------------------------------

------------------------------------------------------------

------------------------------------------------------------

------------------------------------------------------------

------------------------------------------------------------

------------------------------------------------------------

------------------------------------------------------------

------------------------------------------------------------

------------------------------------------------------------

------------------------------------------------------------

------------------------------------------------------------

------------------------------------------------------------

------------------------------------------------------------

------------------------------------------------------------

------------------------------------------------------------

------------------------------------------------------------

------------------------------------------------------------

------------------------------------------------------------

------------------------------------------------------------

------------------------------------------------------------

------------------------------------------------------------

------------------------------------------------------------

------------------------------------------------------------

------------------------------------------------------------

------------------------------------------------------------

------------------------------------------------------------

------------------------------------------------------------

------------------------------------------------------------

------------------------------------------------------------

------------------------------------------------------------

--------------------------------

3L dna:chromosome chromosome:AgamP3:3L:11198000:11210000:1

------------------------------------------------------------

------------------------------------------------------------

------------------------------------------------------------

------------------------------------------------------------

------------------------------------------------------------

------------------------------------------------------------

-----------------------------------------------------------T

TACTACGAATTCCAGAGGTGGGTGATAATGATCTAATTTAACAATAGGGTCAATACTACA

AGAAACTGGTGAACATGAATATGTCACTAACTCATTAACAAAAACTAAATCTAGTTGCCT

CCGAAAAATATTTACG-ACCCCTGAAAGTTGACGCAGATTGAATTCAGCTAATCCGTCTA

TAAAGTAGGATGAGGCAGGGTTAACGGCAAGCGGTGCGAAATAATCCTCATTTTGAGACC

ACGATAGTCCAGGTTGATTGAAATCACCGCATAAGACAAACAAATGATTTTTGTTCTACA

CAACATTTTAAAATG--AAATTTGTCATAAATTTGCGTTATGTTGTTAGCTTACAAATAA

GTTTTGATCATATGTTTTTGATCATATGTATATGTTTTGATCAATTTGTTCTTCT--TGG

GTTTGCCGAACAAGTTGCGTTGTCATGCCGGCCTATACAGGCTTTAGAGACTTATTGGGT

ACCACGCATCCGGATACACGGTCCTTTGCTACAAGAGGTAGTCCATACCAGATATGAAAT

CATGAAGGGAATGTTGTAAAGTCGCTGACGACTCAATAACATGCTCTGAAATTGGGAAGA

ACCAATGCCAAAGATGTGCAAATGAAAATAAAATAAATTATGTCAAAGATCCTCGA-ACA

TGTCAAGAATGCCAAAAGATTGCATATAAATTATAAGTTTGATGTTACATTTGTTCAGAA

AAATATTTT--CTGTAGAATGAATAATGTAGGTACTTTATTTTGGGACAGTGATATTTAT

CAATTTTGAAAAAAAAAAAAATGTTTTCTTGAAGCAAACTTCTCATTTTTGTCATGCTAT

GGTACTACACATTCTTAAGCATATCACGAACAATGTTTCAAAAAATAAAGCAGTAC-AAT

AACTTCATTTTTCACGAAAAAAAAAAAAACAAATGTTAAATTGTAAGTAGTGGGCACTTT

ATTTTGCCGGCCACTGTATATCGTATTTGTTAGCTGACAACATAACGCAACTTTATGACA

AGTTTCGTTTTAAAATGATGTCTAGAACGTAAATTATTAGGTTTTCAATCCACGAATT-T

TCAAACTCCCATACAAAAAGTTGGGCTTACTCGTGCAG-----GCGGTCGTAGAAATACG

TCGTTTTTTTTGCGAGTAAGTTACAGTAGAAAACAATATCAAAATTCCAGGAGAATATTG

TTAGTGGAGATGGAAGCATTGATAAAAAAAATATTAGACAAGCAGATAAAAAGGTATG--

AGCTTTTGAATCCTCAAAAATGTAATGTCTCATACAAAATATACGGTCCTACCCAGGTAG

GCGGTCATAGATGTAAAGGGGTTTGGATAGAGTTTGGAAGCACTGAAGATTACTATGATG

CATATCAAAGTAAGTAGTAGCTGGTCTCGCGATACAGTCGTCATCTCGTACGACTTAACA

ACATGCCCATCATGGGTTCAAGCACCGAAT-AGACCGTGCCCCCATACGTAAGACAGACT

ATTCTACTATG-----TGTAATAAGTAAGTCACTGAAAACCGAGCAAAGTAA-AGTAGTG

GCACAGGCACAACGACA---------ATCTTTGCGATAACGGT--TGTTGTGCCAAAGAA

GAAGAAGAAGAAGAAGAAGAAGAAGAAGAAGAAGAAGAAGATATCAAAGT-ATGTCCATT

TTGTCCCGC-GTGCTCATTTTGCCCCGTGTTCCCTTACTCTAGCGGGAATCGT-ATTTGG

TTGAGCTATCTTGCAAATAAGGTCGACCATGCCAGAGTAACATGGGTTCGTAGCGAAGTG

ATCAACTGTTATCAATGTTTTATACGTTTCGCGTTTTCACCTTTGTGT-----ATAATAA

TTGGAATCAAATCATATAAGATGGTTTAGTATGCACTTTCGAGCCAATTTAGTT-TTTCT

TTCATTCAACTAATTCTTGCAGGCTTGGAGTTTAAAGAAAAAGTACTTTAAG-GTATAAA

AAAACCCCAGC-ACAACATAAATAATT--GTAAGTTGTAAGATAGTGATTTGATAAACAT

GAAGCA-AAACTGAAAAACAATTGAAGCAATTTTTCCCAGATAATTATTTATCTTTTTGT

TATTTATTGATTAGAATTCAACAGACCACTGC----CTTCTTAAAGCGAAACAAGGTCAC

AAACATATGATACAGTCTC-GTTTCAGTCATCGTTTTTGCCGCGTTCAAGAAACAAGTGT

CCGTCAAACTTCTTCATCTTCTTTTT---TTATTTCCAAATCAATGGTTAAGGCATTTGC

TGCATTCT--ATTTGCTAACCTTGTACATCATGTACGTATCTGTTCTGCAT--TGATCAA

TTGCGTGGTCCATTTCA-TGTAT--ATTAATCCTTGCCAA-ATAGTTAACCGAATCAAAA

ACACCAATTGAGTCCTTTTCACTTGATGCGTTGATGGATCAATTAACTTTTCGTACTGTT

CGTTTCTAGAGAACTAACGGA--GAACTAATCATGACGTCTTCACACATTACGGCTTACC

ACAGGTAAATATTTCACCCAGTACAATGCAAACGACTCACTAAGCATTGTTTTAAAATCA

TTGTTTTGAACCGTATCACAAGAT-TATTTAAGAAAATTTCTTCAATAAAAAGCTACTCT

TTA-ATTTCGTGTTTACAAATACTTCAGCAGAAGAAATACAGAATTGCAGAAAAATTA-G

CCTGCTCGAAATAACATCAAACTACTAATACTTATGTTTCTATAAGAGTTTACAATGAGC

AAATAACAATATTAAACAAAACTATGTGTGATAAAAGCAATACAGTAATCTATCAGTGAG

CCAAAAAACTTACTATGCAACAAGTTAATGCCATACTGTTTA----ATGGCAACCTAGCA

TAAAAT--TCAAACATTAAATTACTTTTGT-TAAAGGTAAACTCTAGCAGGATGTGCAGT

TTTGCAAGCATGAATACTATTAACGCACTGCAATATTAGCCACTGGAATCGGTATTTCTG

TACAAACCAAGGGGATTTATTCTTATCGCCCGGCGTTCATGCAGGTCGCTATGTTTACAG

TAACATCTGAGGGCAGGGAATCCCCAATAACAACAGCACAGCTTCACCGATAGGGATGCA

AACTGACCGTGATTCAGTTCAATCGTAGACGGCATCCGAAGCAGACGCGAAATCGCACAA

CGTCCAAACCAACCATGTGGCAGTTCATAAGGTCACGAATATTAACGGTGATAATCTTCA

TAGGTGCTGCTCATGGGTAGGAACAAACGTGCTGGAAGTGCTGTGCCAATCGATTGAGTT

GAGAGTAATTTGTACACTACGAAACGAAATATACTTTTTCTAGGCTACTGGTTGTGGGTC

CGAAATTTATACGGGCCAACCAGGAATACACTCTGGTGATCAGCAACTTTAACTCACAGC

TAAGCAAAGTGGACCTGCTGTTAAAACTGGAAGGCGAAACTGATAATGGTTTAAGCGTTC

TGAACGTTACCAAGATGGTTGACGTGCGACGTAATATGAACCGAATGATCAACTTCAATG

TATGAAGAGTGAGCGATATTAGTTTCTAAGGCTTACAACTAAA----ACATTCGATCCTT

TGCAGATGCCTGAGGATCTGACGGCTGGAAACTACAAAATAACTATCGATGGACAGCGTG

GCTTCAGCTTTCACAAGGAGGCAGAGCTGGTGTATCTCAGCAAATCGATATCGGGGCTAA

TACAGGTCGATAAGCCCGTATTTAAACCTGGGGATACGGTGAACTTCCGTGTGATCGTGC

TGGACACGGAGCTGAAACCGCCGGCGAGGGTCAAGTCGGTTTATGTAACTATACGAGATC

CTCAGCGCAATGTGATTCGCAAATGGTCCACGGCAAAACTGTATGCCGGTGTGTTCGAGA

GCGATCTACAGATAGCGCCTACTCCAATGCTCGGGGTCTGGAATATCTCGGTGGAGGTGG

AAGGAGAAGAGCTTGTGTCAAAGACGTTTGAGGTGAAGGAGTACGTGTTGTCAACGTTCG

ACGTGCAGGTCATGCCATCGGTGATTCCACTGGAAGAGCATCAAGCTGTGAATCTTACAA

TCGAAGCGAACTATCACTTTGGTAAGCCAGTGCAAGGAGTGGCCAAGGTGGAGCTGTACC

TAGACGACGATAAGCTAAAACTGAAAAAAGAGCTGACTGTGTACGGAAAGGGCCAGGTAG

AGTTGCG---CTTTGACAATTTTGCAATGGATGCGGATCAGCAGGATGTACCAGTGAAGG

TGTCGTTCGTCGAGCAGTACACAAGTAAGAATCATGTTC---GAGATACCGTTGCTAACA

GTGATTAAATAAGAAATATCTCTTCATAGATCGTACGGTGGTCAAACAGTCACAAATCAC

GGTATATAGGTATGCGTACCGAGTAGAGTTGATAAAAGAGAGTCCACAGTTTCGTCCGGG

ACTCCCGTTCAAATGTGCGCTTCAGTTTACACACCATGATGGAACACCGGCTAAAGGCAT

TAGCGGTAAGGTAGAGGTATCCGATGTACGATTCGAA-ACGA-CAACAACGAGTGATA--

--ACGATGGATTGATTAAGCTCGAGCTGCAACCAAGTGAGGGTACTGAACAACTCAGTAT

TCACGTAAGT-ATCTAGAATGTT-----TAGTTAATGGTTGACAAAGCATCTTAAAGGGT

CAGTTCTTTTGCAGTTC---AATGCTGTTGATGGATTCTTTTTTTATGAAGATGTGA-AT

AAGGTAGAAACGGTTACGGATGCGTATATTAAACTGGAGCTGAAATCACCGTGAGTAATA

ACTCGCTACAAAGTGAAACTGGCAGTGTGATGTATAACATAACATA--TCGTTCTAGCAT

CAAACGGAACAAATTGATGCGTTTCATGGTGACGTGCACGGAGCGCATGACATTCTTCGT

GTACTATGTCATGTCAAAGGGCAATATCATCGATGCAGGATTCATGCGACCCAACAAGCA

ACCGAAGTACCTGTTGCAGCTGAACGCAACAGAAAAGATGATTCCGAGGGCGAAAATTCT

CATCGCTACCGTAGCGGGCCGCACGGTGGTGTACGACTTCGCAGACCTCGATTTCCAAGA

GCTTCGCAATAATGTAAGC-----ATTTGTTTGTC-----GTGTTGTTTAACGTAACA--

CTTATTCATGTTG-TGTGGAAACAGTTTGATTTAAGCATTGACGAGCAAGAGATCAAGCC

GGGACGACAAATCGAGCTGAGCATGTCTGGACGCCCAGGAGCGTACGTTGGGCTGGCCGC

GTATGACAAAGCCTTGCTGCTTTTCAACAAGAACCACGACCTGTTCTGGGAGGACATTGG

GCAGGTGTTTGATGGGTTCCATGCAATCAATGAGAACGAGTTTGACATATTCCACGTATG

TATGATGCGAAAATCGAGCAAGAGATATCAGAAAAC-AATTATCAAAAACGAGACGCG--

TAATTATTTTTGCAGAGCTTGGGTCTGTTCGCCAGGACATTGGACGATATCTTGTTCGAC

AGTGCAAATGAAAAGACGGGGCGTAATGCACTGCAGTCAGGCAAGCCGATCGGCAAGCTG

GTGTCGTATCGGACGAACTTCCAGGAATCGTGGTTGTGGAAAAATGTTTCCATCGGACGA

TCGGGAAGTCGCAAGTTGATCGAGGTAGTACCGGACACGACCACCTCCTGGTATCTGACG

GGCTTCTCGATCGATCCCGTGTACGGGTTGGGTATCATCAAGAAGCCAATCCAGTTCACA

ACAGTCCAGCCGTTCTACATCGTAGAGAACTTACCATATTCAATCAAACGAGGCGAAGCG

GTTGTGTTGCAGTTTACGCTGTTCAACAACCTTGGAGCGGAGTATATAGCCGATGTGACG

CTGTACAATGTGGCCAACCAGACCGAGTTCGTCGGACGTCCAAATACGGGTGAGTGTGGT

TTACATCAATCAACCCTTGATTATTGAAAACTTCAACATTAATTTTATGTTCAGATCTCA

GCTACACCAAATCCGTGAGCGTTCCTCCAAAAGTTGGTGTGCCAATCTCGTTCCTCATCA

AGGCCCGCAAGCTCGGCGAGATGGCGGTTCGTGTAAAGGCTTCGATAATGCTGGGACACG

AAACGGACGCCCTGGAAAAGGTAATACGGGTGATGCCTGAAAGTTTGGTGCAGCCGAGAA

TGGATACACGCTTTTTCTGCTTCGACGATCACAAAAATCAAACGTTTCCGATCAACTTGG

ACATCAACAAGAAGGCCGACAGTGGATCGACAAAGATTGAGTTTCGACTAAATCGTAAGT

AGAGGGTGTGAAAGTT-GTGAAAGGAGTTATTGAGAGTTTTTTTCTTCTTTTTACCCAAC

CATTTCTGCAGCCAATTTGTTGACCACGGTCATCAAGAACCTGGACCATCTTCTCGGCGT

TCCGACGGGATGTGGTGAGCAGAATATGGTCAAATTTGTTCCCAACATTTTGGTACTGGA

TTATTTGCATGCCATCGGGTCGAAAGAACAGCATCTAATCGACAAAGCTACGAATTTGTT

GCGTCAAGGATATCAAAACCAGATGCGCTACCGTCAGACGGATGGTTCATTTGGTTTGTG

GGAGACTACTAATGGTAGCGTGTTTCTCACCGCGTTCGTTGGCACATCGATGCAAACTGC

AGTAAAATACATAAGCGATATTGATGCAGCAATGGTGGAGAAGGCATTGGATTGGTTAGC

CTCGAAGCAGCATTTCTCGGGACGGTTTGACAAGGCCGGTGCAGAGTATCACAAAGAAAT

GCAAGGAGGGTTGCGCAATGGTGTGGCCCTCACATCATATGTGTTGATGGCATTGCTGGA

GAATGACATTGCCAAAGCAAAGCACGCAGAGGTGATTCAAAAAGGAATGACCTATCTGAG

CAATCAGTTTGGATCCATCAACAATGCATACGACCTATCGATAGCAACCTACGCGATGAT

GTTGAACGGACACACCATGAAGGAGGAGGCACTCAATAAGCTGATTGATATGTCTTTCAT

TGATGCTGATAAAAACGAACGGTTCTGGAACACAACGAATCCAATAGAAACCACCGCATA

TGCTCTGCTGTCGTTTGTGATGGCCGAGAAGTACACAGACGGTATACCGGTCATGAATTG

GTTGGTGAATCAACGTTACGTTACCGGTAGCTTTCCGAGCACGCAAGACACGTTTGTGGG

GCTGAAAGCGCTGACCAAAATGGCGGAAAAGATATCTCCGTCCCGAAACGACTACACCGT

TCAACTGAAGTACAAGAAGAGTGCAAAATACTTCAAAATAAACTCGGAGCAAATTGATGT

GGAAAACTTCGTGGATATACCGGAGGACACAAAAAAGCTCGAGATCAATGTGGGGGGCAT

TGGATTTGGGTTGTTAGAGGTGGTTTATCAATTTAATTTGAATCTCGTCAACTTTGAGAA

TAGATTCCAACTAGACCTGGAGAAACAGAACACAGGCTCTGACTACGAGCTGAGGCTGAA

GGTCTGTGCCAGCTACATACCCCAGCTGACCGACAGACGATCGAACATGGCACTGATTGA

GGTAACCTTACCGAGCGGTTACGTGGTTGATCGCAATCCGATCAGCGAGCAGACGAAGGT

GAATCCGATTCAGGTAAGAATATTTGAATGTTGAATATCCAGAGCAGTTTGAGCTGACTA

TTATGTATTTACTTTTGATTGCATTCACAGAAAACTGAAATCCGTTACGGTGGCACTTCA

GTCGTTTTATACTACGACAATATGGGCAGCGAGCGTAACTGTTTCACCCTGACCGCGTAC

AGACGCTTTAAGGTCGCATTGAAGCGTCCAGCGTATGTGGTTGTGTATGATTATTATAAT

ACAAGTGAGTAGTAGTCATAGATTGGCTATGGAATTGCACAGGGAATG----------TA

ACACCCGTTGCTTTTTAAATTATTTACAGATCTGAACGCCATCAAAGTGTACGAAGTGGA

CAAGCAGAATTTGTGCGAAATCTGTGACGAAGAAGACTGTCCTGCAGAGTGCAAAAAATA

GAGACTGAACGGTGAAAGGGAAAATATTTTTAAAATGTCGTTAAAGGCAGCTTGAATCGC

AGATACATGCAAGAGTTTA-AATAGTTTTTG-CAAGAGCAAGCATATTAAATAAAGCGCT

TCGTAATGAAGCAAGAACCAATTATTTTACGATTTGCTTTCAAGTTAATAACTGATCTTC

GTAAT--AGTTCATAGATAGTTCTTAAAGAAAAATGT-------TGCAATTTTCGCCAAC

GGTGTTTTTTTTGCATTTAAAATACACACACTTTTTTTGCAAATCTGTGACTTGTTTCTT

TGG---TGGATTTTCTGCTCCTCCTTGCAT--GCTCTTAATTGGTTAGGAATCAGCACGC

GCTGTTCCGATTCC-AGATCTTCGAC-CTGGCGCGCGAACCCTGACCG--CTTCGAACAT

TTAT------TTTCTCTTTCTCTCTCTCTCTCTCTCTCTCTCTCTCTCTCTCTCTTTCTC

TCTCTCTCTGTCTCTCTCTGTCTGTCTCTCTCTCTCTCTCTCTCTCTCTCTCTC-TCTCT

CTGTAGCTGTGATCGTTTTTTATGGCACCGTCGATCAGCTAATCGTTTTAATCGATAAGC

GCACCTGCCTTTACCACTATCTCGCACTTGTTATCATTGTCTCTCTTAA-GTCAAAATAC

GTCTCAACAATTCTAGTATGAATGACGAAAATTAAACTGGCGGAGATATTTTTCTTGGGT

GCCTAAGCTCAACTTTTAAAGGAACGTAAGCTTATGTATGGGTTGAATGTACCATGGACT

CATAAACAATCACTAGAAGCTCTCAGTGCTACACTCAAATATCTTTGAGGCAAAGAT-AA

ATAGAAGGGAGTGATTTAGTACTTCTTTGCGTAGACCTCCGCCAAACT-TAACCAGTTTG

TCCTAGATCAACACAAGCTGATGAAAGAAATGCATGCATAAAGA--CATCATGGATATGG

AAGCTTGTTGAACCTATTTATTTGAAGCA--AAATATAAGAGTAAAAACAGATACTTGTA

GTTCTCAAACATTTTTTCATAAATGTTTAGCAATCGG--TGA----GGGAAAATTATATC

TAGATGCATCTGAAACGCTTAAATTATCATCAAGTTTAA-GTATCTTGGAGCCTAGCTGG

CTAGTATCGCAATACACCAAAATATCTATACCATATTGAAAGAAAGGGGTTAAAAAAGAG

AGCTTTTC----TTGCACCTAATATTACGAATTTCATTAAATTAAAGTAAAGGTTTGGGC

CTGCGGCTTCGAGATAGTATCACTTTAGGAGTTGCGCTGGAAATGTCTAATGGTGCGCAA

GAAACGCAGCAAC-TGCACGGTCTACCA---GAGAGGTGGAGAGAC-GCGCAAGCTCGGT

ACTGCGTTCATGATGCTGGGTG-CGATGCAACAGAGGGTG-ATCGGGTGGTGGCCGGTCA

ACGAACGGATGTGCAGGTTGAGGATTCGTG-GCAGATTCA--TCAACCTGAGCATTATTG

ATGACCACAGTCCGCATTTTAAGAGCACCCATGACGAACAGTAGCCATTTTAT-ACGC-A

GTTTGAGAGGGA----GTACGATCGCTGCCTAAAACATGACGTAAAGATTGTCATAGGAG

ACGTTAACGCTCAGGACGGACAGGAGGAGGCATATAAACCAACCATCGGAAGCTTCAGCG

TCTACCAGCGGACAAACCAAAACGACCTCATGCTCATAAACTTTACCTCGTCCAAGCACA

TAAGCATCCGCAGCACCTTCTTACAGTATGCTCACCGCCGTAGTTACACCTGGAGACAGC

CTAGAGGTGATTCAAAGTCCCAAATCGACCATGTTTTGATTGACGGAAG-GCTCTCGGGT

----ATTATCGACATCAAAACGTATAGAGGTCCGGCACGGTTTGTTTTGGTTGATACAAT

GTTAGCATAGAATAAAAATAAATTGATGGAATTTTGTGGCTTTTTCTTAAACAAAAAACG

ATATCAACTTTTTTCATTTGAGAAGATGATTTTTGAATACGTTTTGTTTGTTTTTTTTAT

GATGAATCAAAACAAACCGTACCAGATCTGACAGCTATTCAATCAAAGTCGACAATTATT

TAAATCATGTGTGTGTTTCAGGTCCTACTCAACACGTCATGCAGAATGGAAACTTTTCAT

TAAGTGTTTGGTACGTTGGGCATTGACACCATAAAACAAAATCTATAGGGTAAATGTAGG

GTAAATGTAAGCAGAGTTTAAGTACCGTTGCTCGAAAGTGAAAATTATGCTAGATAAATA

TATGGAGGACACCGTTGAATGTTAAAATGTTAAGTAAGAATATTATGTACCATATCCCAT

TTGCTGTGGAACAGTCACTGCCGAAATAAAACAGTACAATGCTTTCATGTACAAAATACA

TCTAAGTGGAATATTGCAAATTAGCTGAAAAAAATATTATATTATAATATATTAATT-AT

TATAAATATTATAGTATACTAGCTGGCCCGA--CAAACTTAGTTATTGAAAAAAAAAAAT

TCCATTNNNNNNNNNNNNNNNNNNNNTATCGTGCCCGTATCCCCTCAGGATCCGATCATC

GACTGTAAACCGCCAGGCACCTCACACCAAGTGTCAAAGTGCCGTATACAACACAACAAC

AATCCTGCTCTTTGTATGGGAGTTAGAAAATCATTATTAAATTAAGTTTAGTGCATCATC

TGAACGATTTTTAAGTCTTAAAACCTATTCTCATACCAC-CATAAGGTATCTGCAAAGTT

TCGTTCAAAATGATCCAGCCGTTTCGGAGCTAGCTCGCAACAAACACCGTGACACGAGAT

TTATATATATATATATAGATAAATTATGCTGTAGTGTTATTTTTTGCTGTAGTCGTCGAG

GCAATTGGTCCATTGAAATACGACACTATCCATCAAGTACCTTAAAAGAGATGTGTGTCC

GCGGCTAATCAATAGACTTTTCGTTGTATCACAATATAAACAAACAGGAGACAGGGTTTC

TTCAATTAGATAAAATAGTATCTCAGTTGACTTCATAAATATAACTAACATAGCATAAAA

AAATGTATTCCA---AAGCTTTGTTTTTTTTTTAATCTGTAGAAATAGCCATATGTGGTC

TATATCTGAAAAAAACTACTCATTGGCATGTATTTTAGTGATTTGATTGGTGTACGCATA

GTATTCTGAATAGCGGGGACTCGATCCACTCAATATTTGAGCAAATGACAGGTATTCTTT

TGATG--AATTTATTGAAGAAATAATATAATTCCAAATTATCCATATTGTGTAATAATAT

AAATCAATCTGT--TACTGGAATGTGGAATAGCGATTCGATGCCTTGTGAGTGGAATCTC

GGCATTCTACCCGATATACAAAGATGGACTGCAACAACTATAGTGGTAGCACGGTGTTCA

ATACCACCTATAAAATACTCTCCCTGATCTTTCAGGATCGTCTAGTCCCG-CACGTCGAA

GAGATAGTTGGAAACTATCAAAGAGGTCTTCACCATACGGCAGATCTTAGAGAAGATAGC

TGAATACAGATACAACACATACCATCTCTTAATTGACTTCAAAGCCGCATATGATAGCAT

AGCCAGGTTTAAACTGTATGACGCTATGAGCTCTTTTGGAATCCCAGCCAAACTGATAAG

GCTAGTTAGAATGACTATGATCAACTAGCCAGGT-GAAGGAGGATGGAAAACTCTCAGGT

CCTTTTGCTATCACCAAAGGACTGCGCCTGGGGCACGGGCAGCTCATTTTTGTTTTAAAC

CTTTCGTCTTGTTTTGTTCAAAGAACATCATACGTTCAGAAGATATTGTTTAAATCAAAT

ATCAACAGTACGCGATTCCTTCTTCATTGGTGCAACAACCGGAATGAGCTATCAGTGACT

TATTGATTATCCATAGCAGGA-TAATCAGTCCTATGTATGTGGGGCATGGTCAATACGTG

GCTTGAAACCTTGACGGGCATGTTGTTAAGCCGTGCATATTGACGAGAGTGCCACAGGAA

AGGTCATTTCTCGCGATACCTATGCGATTACGCGATTCGTGGTGTACACACATTTGCAGT

ACGACAATATTTCTCAGTGGACTTTTTGACATCTCCGTAAACTCCTAAACTTCGGTAAGT

AATATTGTGCAGTGGATCTTATATGAATGATC

**References**

**1. Holt RA, Subramanian GM, Halpern A, Sutton GG, Charlab R, Nusskern DR, Wincker P, Clark AG, Ribeiro JMC, Wides R *et al*: The genome sequence of the malaria mosquito *Anopheles gambiae*. *Science* 2002, 298(5591):129-+.**

**2. Baxter RHG, Chang C-I, Chelliah Y, Blandin S, Levashina EA, Deisenhofer J: Structural basis for conserved complement factor-like function in the antimalarial protein TEP1. *Proceedings of the National Academy of Sciences* 2007, 104(28):11615-11620.**
